# Mutagenesis-based optimal design of plant peptide phytosulfokine for enhanced biological activity

**DOI:** 10.1016/j.csbj.2025.03.029

**Published:** 2025-03-24

**Authors:** Rui Ye, Chen Xu, Zhong-Jie Ding, Shao-Jian Zheng, Siewert-Jan Marrink, Dong Zhang, Ruhong Zhou

**Affiliations:** a Institute of Quantitative Biology, School of Physics and College of Life Sciences, Zhejiang University, Hangzhou, Zhejiang 310058, China; b The First Affiliated Hospital, College of Medicine, Zhejiang University, Hangzhou, Zhejiang 310058, China; c Groningen Biomolecular Sciences and Biotechnology Institute, University of Groningen, Groningen 9747 AG, the Netherlands

**Keywords:** Phytosulfokine, Analogs design, Molecular dynamics, Free energy perturbation, Mutagenesis

## Abstract

Recognition of phytosulfokine (PSK), a sulfated pentapeptide, by its receptor PSKRs is crucial in regulating plant growth, development, and reproduction. However, designing highly active PSK remains a formidable challenge due to the lack of understanding of the structure-property relationship, structural dynamics, and the binding characteristics of PSK. Here, with a combined theoretical and experimental approach, we have investigated the binding dynamics of key interactions between PSK and AtPSKR1^LRR^ to reveal the molecular mechanism of PSK recognition. Our molecular dynamics simulations and free energy perturbation calculations demonstrate that the sulfated tyrosines (PSK^sY1^ and PSK^sY3^) are indispensable for forming stable PSK-AtPSKR1^LRR^ complex, while the alanine substitution at PSK^Q5^ site is rather tolerated. Furthermore, two promising PSK peptide analogs (PSK^Q5A^ and PSK^Q5K^) with enhanced biological activity have been designed through *in silico* mutagenesis studies and *in vivo* experiments. They have a strong promoting effect (20 % enhancement) on stimulating root development compared with the wild-type PSK treatment. This work offers an effective strategy to design new peptide-based drugs for facilitating plant growth and consequent crop productivity, potentially benefiting efforts to address the global food crisis.

## Graphical Abstract

**Figure:**
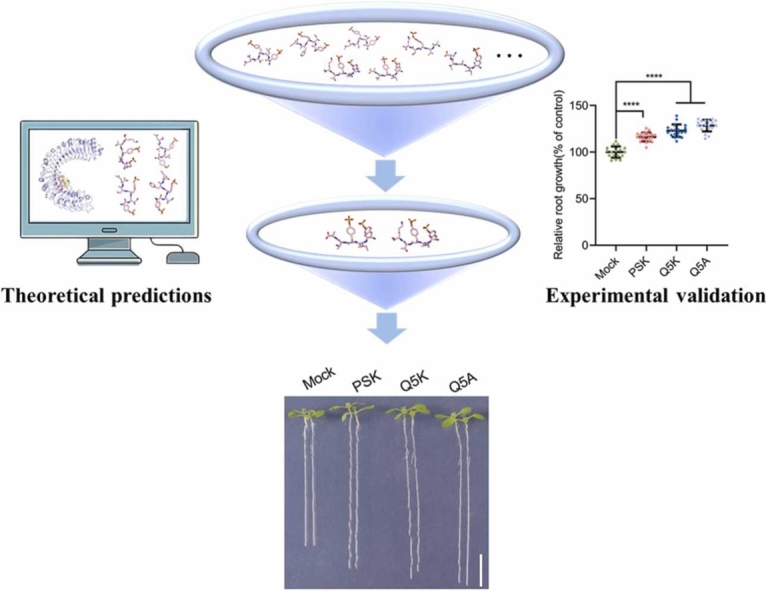
Strategies for designing PSK analogs with enhanced bioactivity.

## 1 Introduction

Intercellular signaling mediated by peptide hormones is a crucial mechanism by which plants regulate their growth, development, and reproduction [1], [2]. These phytohormones also play an important role in environmental responses that are essential for plant survival and adaptation to changing conditions [3], [4]. A notable example is the sulfated pentapeptide (Tyr(SO_3_H)-Ile-Tyr(SO_3_H)-Thr-Gln) phytosulfokine (PSK), which is the first peptide growth factor discovered in plants [1]. PSK is generated from the COOH-terminal region of an 80-amino acid precursor protein, which is widely distributed in higher plants [5]. The biosynthesis of PSK involves tyrosine sulfation of the precursor protein by a tyrosylprotein sulfotransferase (TPST) enzyme in the cis-Golgi apparatus, followed by proteolytic cleavage of the sulfated precursor in the apoplast [6]. Then, mature PSK peptides are secreted from individual plant cells in response to changes in the levels of auxin and cytokinin, acting as autocrine-type growth factors to regulate cellular dedifferentiation and proliferation in plants [7].

Once secreted, mature PSK peptides are recognized by membrane-bound PSK receptors (PSKRs) on the surface of plant cells. Since first being identified in *Daucus carota* (carrot), known as, DcPSKR, PSKR has been found to conserve among different plant species [5]. For example, *Arabidopsis thaliana* expresses two different PSKR orthologues: PSKR1 (AtPSKR1) and PSKR2 (AtPSKR2), with the PSK sensing occurring mainly through PSKR1 [8], [9]. AtPSKR1 contains three main domains: an extracellular domain with leucine-rich repeats (LRRs), a transmembrane domain, and an intracellular kinase domain (KD). The extracellular domain of AtPSKR1 (AtPSKR1^LRR^) contains 21 tandem copies of LRR with an island domain (ID) [5], [8]. Previous studies indicated that PSK first binds to AtPSKR1^LRR^ and then leads to the activation of AtPSKR1 kinase activity via Ca^2+^/CaM (calmodulin) binding [10], [11]. Subsequently, the PSK-AtPSKR1 complex interacts with Brassinosteroid Insensitive 1 (BRI1)-Associated Kinase I (BAK1), a member of somatic embryogenesis receptor-like kinases (SERKs) that generally act as a coreceptor with other leucine-rich repeat receptor-like kinases (LRR-RLKs), to activate downstream signaling pathways [12]. Together, the PSK-AtPSKR1-SERK1 complex activates a signaling pathway that regulates various cellular processes, including cell proliferation, differentiation, and growth [13], [14]. Recently, Wang and co-workers [15] solved the crystal structure of the extracellular LRR domain of PSKR1 bound to PSK in *Arabidopsis thaliana* and revealed the structural basis for PSKR recognition of PSK and allosteric activation of PSKR by PSK. However, the structure-property relationship, the structural dynamics, and the role of individual residues on the binding interaction between PSK and AtPSKR1 remain largely elusive, which hinders further efforts to find PSK-like peptide hormones and design PSKR-specific small molecules.

**Scheme 1:**
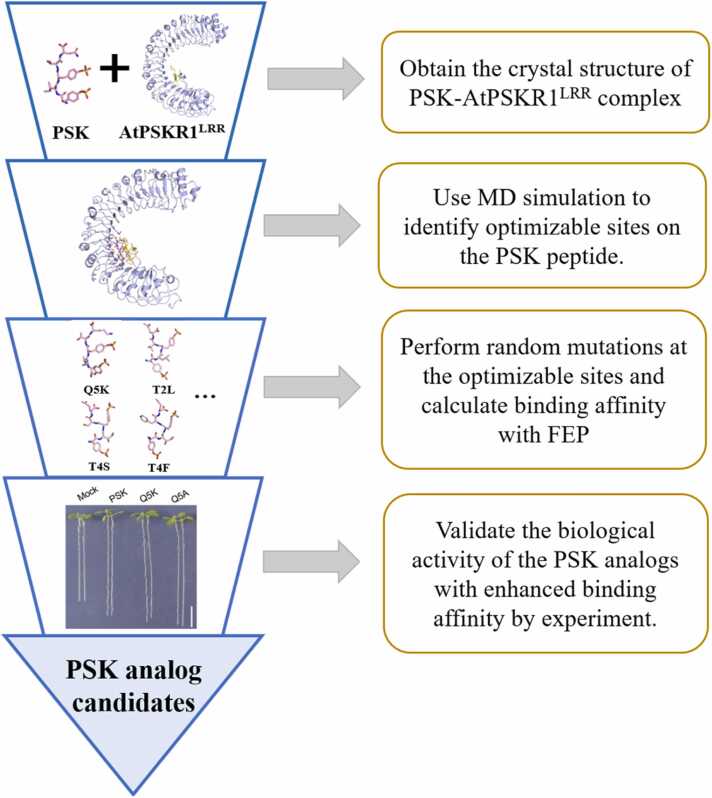
Strategies for designing PSK analogs with enhanced bioactivity.

In this work, we employed atomistic molecular dynamics (MD) simulations and free energy perturbation (FEP) calculations to characterize the detailed recognition mechanism of PSK by the AtPSKR1^LRR^ domain and to design PSK-like peptide hormones with enhanced activity. An effective strategy for designing highly bioactive PSK analogs was meticulously developed (Scheme 1). Starting from the crystal structure [15], the binding dynamics between PSK peptide and AtPSKR1^LRR^ domain were studied firstly by MD simulations. The applicability of FEP method in PSK-PSKR^LRR^ binding complex was verified and then used to assess the contributions of individual residues of PSK in directing its binding to AtPSKR1^LRR^. Then, *in silico* alanine-scanning of PSK and desulfated mutations at PSK^sY1^ and PSK^sY3^ were performed to assess the role of individual residues in binding affinity. Subsequently, the PSK peptide analogs were designed to enhance the binding affinity through *in silico* mutagenesis studies. Finally, the biological activities of designed PSK peptide analogs were validated by plant *in vivo* experiments. Our study provides a promising strategy to rationally design PSK peptide analogs with enhanced activity and specificity for the PSK-PSKRs^LRR^ signaling pathway and opens a new avenue for the development of more effective plant growth modulators to improve crop yield.

## 2 System and method

### 2.1 Molecular dynamics simulations

**Fig. 1:**
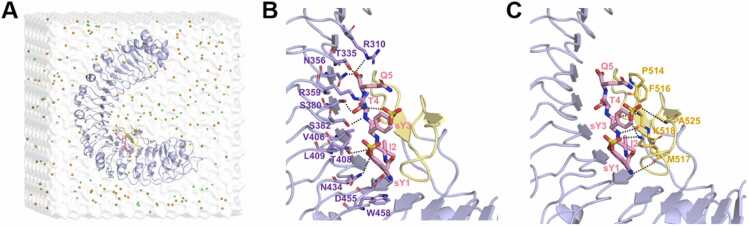
Overview of the binding complex for PSK peptide and AtPSKR1^LRR^ domain (PDB ID: 4Z63). (A) Initial configuration of the simulation system. The PSK peptide and AtPSKR1^LRR^ domain are shown in pink stick and purple cartoon representations, respectively. The island domain of AtPSKR1^LRR^ (AtPSKR1^ID^) is colored in yellow. Water is shown transparently for clarity. Sodium and chlorine ions are shown as orange and green spheres, respectively. (B) Detailed interactions of PSK peptide (pink) with the inner surface of AtPSKR1^LRR^. The key residues are shown as purple stick. sY, sulfated tyrosine. (C) Detailed interactions of PSK peptide with AtPSKR1^ID^ domain. The key residues are shown as yellow stick. For clarity, oxygen, sulfur and nitrogen atoms are specifically shown in red, tan and blue, respectively.

We performed all-atom MD simulations on the complex of the wild-type (WT) and mutated PSK peptides bound to the LRR domain of AtPSKR1 (AtPSKR1^LRR^, Fig. 1A). The crystal structure of AtPSKR1^LRR^ bound to a WT sulfated pentapeptide PSK (protein data bank (PDB) ID: 4Z63) [15] from *Arabidopsis thaliana* was adopted as the initial structure. The complex was placed in a 10 × 10 × 10 nm³ water box using the TIP3P water model [16], ensuring at least a 1 nm between the protein surface and the box walls. Na^+^ and Cl^-^ were added to neutralize the systems and reach the final ion concentration of 150 mM. All systems were relaxed using energy minimization of 20000 steps and equilibrated for 10 ns, and followed by three independent production runs in NPT ensemble. The simulation time lengths for each system are summarized in Table 1. MD simulations were carried out with GROMACS 5.1.4 [17], using the CHARMM36 force field [18]. Specifically, the energy parameters for sulfated tyrosine (sY) residue were generated using the CGenFF webserver with default settings, and the partial charges were automatically assigned by charge increment fitting scheme adopted by CGenFF [19], [20] (for details, see Table S1). Long-range electrostatic interactions were calculated using the particle-mesh Ewald (PME) method [21], and van der Waals (vdW) interactions were calculated using a cutoff distance of 1.2 nm. The room temperature T = 300 K was controlled using the velocity-rescaled Berendsen thermostat [22]. The pressure was set to 1 atm using the isotropic Parrinello-Rahman barostat [23]. The covalent bonds involving hydrogen atoms were constrained at their equilibrium values by the LINCS algorithm [24], which allows a time step of 2 fs. The snapshots were rendered by the PyMOL program [25].

**Table 1:** Summary of the MD simulations performed in this study.

Systems	MD Time	No. of repeats
PSK-DcPSKR^LRR^	1000 ns	3
PSK-AtPSKR1^LRR^	1000 ns	3
PSK^sY1A^ -AtPSKR1^LRR^	700 ns	3
PSK^I2A^ -AtPSKR1^LRR^	700 ns	3
PSK^sY3A^ -AtPSKR1^LRR^	700 ns	3
PSK^T4A^ -AtPSKR1^LRR^	700 ns	3
PSK^Q5A^ -AtPSKR1^LRR^	700 ns	3
PSK^sY1Y^ -AtPSKR1^LRR^	700 ns	3
PSK^sY3Y^ -AtPSKR1^LRR^	700 ns	3
PSK^Q5K^ -AtPSKR1^LRR^	700 ns	3

### 2.2 Binding free energy change calculations

The relative binding free energy (binding affinity) changes were carried out with the FEP method. We calculated the free energy changes for the same mutation(s) in both the bound state (AtPSKR1^LRR^+PSK peptide) and the free states (PSK peptide only) with 6 replicas for each calculation. FEP calculations were performed similarly to our previous work [26], [27], [28], [29], [30]. The mutation’s binding free energy change Δ*G* can be calculated as follows:

(1) Δ*G*_*λ*_ = −κ_B_T*ln*〈*exp*(−β[V(λ + Δλ) − V(λ)])〉λ,

(2) Δ*G* = ∑_λ_ Δ*G*_λ_,

where V(λ)= (1 −λ)V_1_+ λV_2_, with V_1_ and V_2_ representing the potential energy of the wild-type and the mutant, respectively. The parameter λ changes from 0 to 1 as the system transforms from the wild-type to the mutant. Multiple independent FEP calculations were carried out using a starting structure chosen randomly from the corresponding MD production run. We used a 46 window strategy with soft-core potential (λ = 0.00, 0.00001, 0.0001, 0.001, 0.01, 0.02, 0.06, 0.1, 0.14, 0.18, 0.22, 0.26, 0.30, 0.34, 0.38, 0.42, 0.46, 0.5, 0.54, 0.58, 0.62, 0.66, 0.7, 0.74, 0.78, 0.82, 0.86, 0.885, 0.89, 0.891, 0.893, 0.895, 0.898, 0.9, 0.911, 0.913, 0.915, 0.918, 0.92, 0.925, 0.94, 0.98, 0.99, 0.999, 0.9999, 0.99999, 1.00). Quantifying absolute binding affinity is difficult due to the lengthy period of the binding process between AtPSKR1^LRR^ and peptide. To address this, we estimated the relative binding free energy change ΔΔ*G* using a thermodynamic cycle (Fig. S1). Instead of calculating the direct binding energies Δ*G*_*B*_ and Δ*G*_*A*_, we obtained the free-energy changes for the identical mutation in both the bound state (AtPSKR1^LRR^ -PSK, Δ*G*_*2*_) and the free state (PSK, Δ*G*_*1*_). The overall change in free energy should be zero according to this thermodynamic cycle, and the relative binding affinity for the mutation from A to B is:

(3) ΔΔ*G* = Δ*G*_*B*_ − Δ*G*_*A*_ = Δ*G*_*2*_ − Δ*G*_*1*_

The simulation time for each run was 18.4 ns, amounting to 220.8 ns (18.4 ns × 6 runs × 2 states) of FEP calculations for each mutation.

We noted that charge changes caused by residue mutations in FEP calculation might introduce errors in estimating relative binding free energy changes, particularly due to the finite size effects associated with the periodic boundary condition and the use of PME method to calculate electrostatic forces. To address this, based on the previous study [31], we constructed a sufficiently large simulation box (10 × 10 × 10 nm^3^) with a solvent buffer larger than 1 nm and used an ionic strength close to the experimental condition to minimize the occurrence of electrostatic finite-size artifacts. In addition, we also performed FEP calculations on the representative sY1A mutation using different simulation box sizes for further validation and observed consistent results (see Fig. S2A-B). Furthermore, we also observed good agreement of relative binding free energy changes between experimental measurements and FEP calculations (see below for details). Taken together, these results indicated that the setup of our FEP calculations is reasonable.

Following the procedures used in our earlier work [32], [33], [34], we performed the decomposition of binding affinity into electrostatic and van der Waals components to account for the contribution from intermolecular interactions involved in the peptide binding. In this investigation, we employ a straightforward decomposition through the FEP formulation by accumulating electrostatic and van der Waals contributions respectively, *i.e.*, V(λ)=V(λ)_elec_+V(λ)_vdW_, in the same ensembles as the full interactions in Eq. (1). As the decomposition of free energy changes is path dependent [35], [36], [37], [38], the binding free energy ΔG is not purely additive from the two components, leading to a minor coupling term due to nonlinearity of the formulation [37]. Free energy components for the bound state (AtPSKR1^LRR^-PSK complex) and the free state (PSK) were averaged over all six replicas. NAMD [39] with the CHARMM force field and TIP3P water molecules were performed for FEP calculations. The error bars associated with free energy changes were calculated as standard errors.

### 2.3 Mass spectrometric analysis

The mass spectrometric analysis was performed by Chinese peptide Biochemical Co. Ltd. (Hangzhou, China). The molecular weight for WT PSK [Tyr(SO_3_H)-Ile-Tyr(SO_3_H)-Thr-Gln] peptide is ∼846.8. The molecular weights for PSK^Q5A^ [Tyr(SO_3_H)-Ile-Tyr(SO_3_H)-Thr-Ala], and PSK^Q5K^ [Tyr(SO_3_H)-Ile-Tyr(SO_3_H)-Thr-Lys] variants are ∼789.7 and 846.8, respectively (Fig. S3).

### 2.4 Phenotypical analysis

To assess the promotion of *Arabidopsis thaliana* root growth by different variants of PSK peptides, germinated seeds of WT (Col-0) were grown on 1/2 Murashige & Skoog (MS) agar medium plus 0.5 % sucrose and 0.8 % agar (Sigma-Aldrich, A7002) with or without 100 nM WT PSK or its variants (PSK^Q5K^ and PSK^Q5A^) for 8 days. To assess the promotion of *Brassica napus* (the Westar was used as WT) root growth by different variants of PSK peptides, germinated seeds of WT were grown on the same medium with or without 200 nM PSK and PSKm (PSK^Q5K^, PSK^Q5A^) peptides for 6 days. The root lengths of seedlings were measured by ImageJ software. The PSK response was evaluated by relative root growth (the percentage of root length under related peptides treatment/ root length under control ×100).

To assess the promotion of *Oryza sativa* (the Nipponbare was used as WT) growth by different variants of PSK peptides, rice seeds were germinated for 1–2 d and then transferred to hydroponic container floating on a Kimura B nutrient solution (CM0800, Coolaber) for 4 days. Then the seedlings of WT were exposed to the same nutrient solution contained 0 or 200 nM PSK and PSKm (Q5K, Q5A) peptides for 24 h. Root length was measured at 0 and 24 h with ruler. The PSK response was evaluated by root elongation.

### 2.5 Mature cell length measurements

To analyze the mature cell length of Col-0 with WT PSK and PSK variants treatments, we employed the seedlings from the phenotypical analysis and used fluorescence microscopy to visualize cell outlines stained with Propidium Iodide (PI) which specifically binding to the plasma membrane. The fluorescence signals were detected using a confocal laser scanning microscope (Zeiss). The seedlings mature cells lengths were measured by ImageJ software.

### 2.6 Bimolecular fluorescence complementation (BiFC) assay

For generating constructs for BiFC assay, the full-length PSKR1 and the full-length BAK1 fragments were cloned into pUC35S-nYFP or pUC35S-cYFP (BIOGLE GeneTech) vectors to obtain PSKR1-nYFP and BAK1-cYFP. These plasmids were selectively co-transferred into the *Arabidopsis* mesophyll protoplasts. Cells were incubated in W5 solution for 10–12 h, and the expression of YFP was detected using a confocal laser scanning microscope (Zeiss). For WT PSK and PSK variants treatment, cells from same pool were treated with 2 µM WT PSK or PSK variants for 10 min before fluorescence detection. The nYFP or cYFP empty plasmids were used as negative controls.

### 2.7 Split luciferase (LUC) complementation assay

The *35S:cLUC-BAK1* and *35S:PSKR1-nLUC* constructs were transferred into the *Agrobacterium GV3101* lines individually, and the *GV3101* lines were co-infiltrated into the *N. benthamiana* leaves. After infiltrating 48 h, the *N. benthamiana* leaves were infiltrated 2 µM WT PSK or PSK variants for 2 h and performed luciferase imaging by using a NightShade LB 985 *in vivo* Plant Imaging System with a CCD camera.

## 3 Results

### 3.1 Dynamic binding between PSK peptide and AtPSKR1^LRR^

From the solved crystal structure [15] of the PSK-AtPSKR1^LRR^ binding complex (PDB ID: 4Z63, see Fig. 1A), we performed three independent 1-μs MD simulations to characterize the dynamic properties of key interactions for the sulfated pentapeptide PSK bound to AtPSKR1^LRR^ domain. The superior stability of PSK-AtPSKR1^LRR^ binding complex was illustrated by the root mean square deviations (RMSDs) analysis (average RMSD ∼ 2.5 Å, see Fig. S4). As shown in Fig. 1B and C, PSK peptide adopts a β-strand conformation, forming an anti-parallel β-sheet with the island domain of AtPSKR1 (AtPSKR1^ID^), and rich hydrogen bond interactions are observed on the binding interface. We further analyzed the contact ratio of each residue of AtPSKR1^LRR^ with PSK peptide during the simulation (Fig. S5). The contact probability is defined as the ratio of the total number of configurations showing contact during the last 100 ns across three independent simulation trajectories to the total number of configurations collected in the last 100 ns trajectories. It showed that 17 key residues (with contact ratio larger than 50 %) in AtPSKR1^LRR^ are responsible for the tight binding of PSK peptide, including AtPSKR1^S380^, AtPSKR1^S382^, AtPSKR1^T408^ and AtPSKR1^D455^ from the inner side of the helical structure and AtPSKR1^F516^ and AtPSKR1^M517^ of the β-sheet, which can form hydrogen bonds with the main chain of PSK peptide. Additionally, AtPSKR1^R310^, AtPSKR1^T335^ and AtPSKR1^N356^ form hydrogen bonds with the free carboxyl group of PSK^Q5^ whereas AtPSKR1^F514^ and AtPSKR1^F516^ tightly pack against PSK^Q5^ (Fig. 1B and C). The sulfated tyrosines contribute to PSK-AtPSKR1^LRR^ complex interactions via both hydrogen bonds involving AtPSKR1^K518^, AtPSKR1^R359^, AtPSKR1^A525^, and AtPSKR1^N434^ and van der Waals packing involving AtPSKR1^L409^, AtPSKR1^W458^, AtPSKR1^K518^, and AtPSKR1^F516^.

Most interestingly, we found that the hydrogen bond network formed by the two sulfate moieties of the PSK peptide with AtPSKR1^R359^, AtPSKR1^K518^ and AtPSKR1^N434^ is dynamic, which is indicated by atom pair distances between PSK^sY1^/PSK^sY3^ and these three residues (see Fig. S6). The N-terminal sulfated tyrosine PSK^sY1^ interacted with AtPSKR1^N434^ stably with an average heavy atom distance of 4 Å. Moreover, PSK^sY1^ also exhibited intermittent contact with AtPSKR1^K518^. For the C-terminal PSK^sY3^, a contact with AtPSKR1^K518^ was formed (distance below ∼ 5 Å) in most of the simulation time. In addition, another dynamic interaction between PSK^sY3^ and AtPSKR1^R359^ (formed at ∼4 Å and disconnected at ∼8 Å, see Fig. S6 and S7) was found. This dynamic interaction network (AtPSKR1^N434^ -PSK^sY1^ -AtPSKR1^K518^ -PSK^sY3^ -AtPSKR1^R359^) connects the anti-parallel β-sheet and inner side helical structure of AtPSKR1^LRR^, which may serve as a modulator to sense and capture PSK peptide. Furthermore, we also calculated the per residue contact area ratio of PSK peptide with AtPSKR1^LRR^ (see Fig. S8), which is defined as the ratio between the residue surface area in contact with AtPSKR1^LRR^ and the total solvent-accessible surface area (SASA) of a particular PSK’s residue. Compared to the other three residues, the residues PSK^I2^ and PSK^T4^ are nearly fully buried and maintain high contact area ratios (∼ 1.0) with the AtPSKR1^LRR^. Overall, those conserved and well-organized salt bridges, hydrogen bonds, and hydrophobic interactions should be collectively responsible for the allosteric recognition of PSK peptide by AtPSKR1^LRR^.

### 3.2 Evaluation of binding affinity changes via FEP analysis

**Fig. 2:**
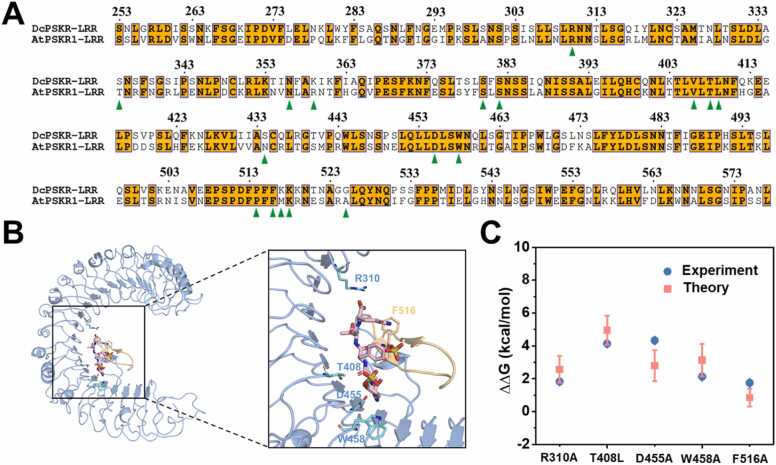
(A) Sequence alignment of extracellular domain of PSKRs from *Daucus carota* (DcPSKR^LRR^) and *Arabidopsis thaliana* (AtPSKR1^LRR^). Conserved residues in two species are boxed with orange ground. Residues involved in recognition of PSK are indicated with green triangles at the bottom. (B) Overall structure of the PSK–DcPSKR^LRR^ bound complex (PDB ID: 4Z5W). The PSK and DcPSKR^LRR^ are shown in pink stick and sky-blue cartoon representations, respectively. The island domain of DcPSKR1^LRR^ (DcPSKR^ID^) is colored in wheat. Five identified key binding residues on DcPSKR1^LRR^ are displayed with stick. (C) Comparison of relative binding free energy changes ΔΔ*G* from FEP calculations (red squares) and experimental measurements (blue circles) for mutations of five key binding residues shown in panel B. The experimental values are derived from previous work [15].

As one of the most rigorous and reliable methods in estimating binding affinity changes, free energy perturbation (FEP) calculation has been successfully used in numerous investigations including protein-protein, protein-ligand, and protein-DNA bindings [26], [27], [28], [29], [30]. Herein, we tentatively investigated the binding free energy changes of PSK-PSKR^LRR^ binding complex from *Daucus carota* caused by mutations in PSKR^LRR^ domain and compared them with experimental results. The sequence alignment of the extracellular domains of PSKRs from *Daucus carota* (DcPSKR^LRR^) and *Arabidopsis thaliana* (AtPSKR1^LRR^) species (see Fig. 2**A**) indicated that the key residues involved in PSK recognition are highly conserved in those two PSKRs. And superposition of the crystal structures for PSK-DcPSKR^LRR^ (PDB ID: 4Z5W) and PSK-AtPSKR1^LRR^ (PDB ID: 4Z63) complexes revealed a consistent binding pattern between PSK and PSKRs (Fig. S9). Here, the missing loop residues 502–511, 518–525 and 622–627 of the PSK-DcPSKR^LRR^ complex were constructed through homology modeling using the PSK-AtPSKR1^LRR^ crystal structure as a template. Of note, the binding affinities (*K*_*d*_) for PSK bound to WT DcPSKR^LRR^ and its five variants (single mutation, Fig. 2B) have been measured in a previous experimental study [15]. On this basis, the experimental relative binding free energy changes caused by mutations can be derived as ΔΔ*G*_*exp*_ = −*k*_B_*T*ln(*K*_*d*_^*wt*^ /*K*_*d*_^*mut*^), where *K*_*d*_^*wt*^ and *K*_*d*_^*mut*^ are dissociation constants for WT and mutated DcPSKR^LRR^ elements, respectively, and *k*_*B*_ is the Boltzmann constant and the room temperature T = 300 K. Therefore, the PSK-DcPSKR^LRR^ binding complex was then treated as a benchmark to test the capability of *in silico* FEP method and the related conclusions could be readily extended to the PSK-AtPSKR1^LRR^ binding system. As shown in Fig. 2C and Table S2, a good agreement between experimental measurements and FEP calculations was observed for the binding free energy changes (ΔΔ*G*) caused by mutations in the DcPSKR^LRR^ domain. Specifically, these mutations all weakened the binding affinity of DcPSKR^LRR^ to recognize PSK peptide, and plants carrying these single PSKR mutants were less responsive to PSK than the WT plants [15]. Therefore, our FEP calculations showed high accuracy in characterizing vital residues and their mutational effects in the PSK-DcPSKR^LRR^ binding system, comparable with experimental measurements, suggesting that its application to the concerned PSK-AtPSKR1^LRR^ complex system appears convincing.

### 3.3 *In silico* mutagenesis study of PSK

**Fig. 3:**
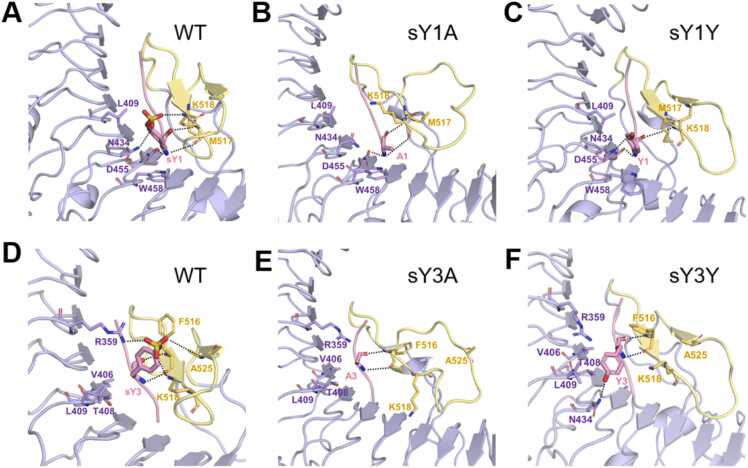
Comparison of structural details for AtPSKR1^LRR^ (purple) bound to different PSK peptides (pink): (A) WT PSK^sY1^, (B) PSK^sY1A^ mutation, (C) PSK^sY1Y^ mutation, (D) WT PSK^sY3^, (E) PSK^sY3A^ mutation, and (F) PSK^sY3Y^ mutation. AtPSKR1^ID^ is colored in yellow.

Subsequently, we employed the FEP method to explore the role of individual residues of PSK in directing its binding with the AtPSKR1^LRR^ domain. The relative binding free energy changes for *in silico* alanine-scanning of PSK peptide were listed in Table 2. For the two sulfated tyrosines at positions 1st and 3rd (PSK^sY1^ and PSK^sY3^), mutations to alanine largely weakened the binding affinity to AtPSKR1^LRR^ (ΔΔ*G* = 4.87 ± 1.16 kcal/mol for mutation PSK^sY1A^ and 4.60 ± 1.34 kcal/mol for PSK^sY3A^), in which the free energy decomposition analysis indicated that the vdW interactions dominate the changes of binding affinity (*∆∆G*_*vdW*_ = 5.26 ± 1.33 and 4.88 ± 1.06 kcal/mol for PSK^sY1A^ and PSK^sY3A^, respectively). To further understand the underlying mechanism of decreased binding affinities, the structural details of mutated PSK- AtPSKR1^LRR^ complexes were studied through additional MD simulations. As shown in Fig. 3, compared with the WT complex (Fig. 3A), PSK^sY1A^ mutant disrupted the hydrogen bond network of the sulfate group with AtPSKR1^N434^ and AtPSKR1^K518^ as well as the hydrophobic interactions with AtPSKR1^W458^ and AtPSKR1^L409^, while the hydrogen bonds between backbone of PSK^sY1A^ and residues of AtPSKR1^D455^, AtPSKR1^M517^ and AtPSKR1^K518^ remained (Fig. 3B). For the PSK^sY3A^ mutant (Fig. 3E), the binding interaction with the hydrophobic pocket (AtPSKR1^V406^, AtPSKR1^T408^, AtPSKR1^L409^ and AtPSKR1^F516^) was greatly weakened due to the absence of an aromatic ring. Moreover, the electrostatic interactions between the original sulfate group and AtPSKR1^R359^, AtPSKR1^K518^ and AtPSKR1^A525^ were also disrupted. In both cases (PSK^sY1A^ and PSK^sY3A^ mutants), the secondary structures of the antiparallel β-sheet of AtPSKR1^ID^ almost disappeared (Fig. S10), and RMSDs of AtPSKR1^ID^ are significantly increased compared with the WT (Fig. S11). These results emphasized the indispensable role of sulfated tyrosines (PSK^sY1^ and PSK^sY3^) in directing the binding of PSK peptide to the AtPSKR1^LRR^ domain, and replacing any of them with alanine largely decreased the binding (hydrogen bond/hydrophobic/electrostatic) interactions and led to the unfolding of the antiparallel β-sheet of AtPSKR1^ID^.

**Table 2:** FEP results (the binding free energy changes ΔΔ*G*) for alanine-scanning and desulfated mutations of the PSK peptide in the PSK-AtPSKR1^LRR^ binding complex. sY, sulfated tyrosine.

Mutation	ΔΔ*G* ($\mathrm{kcal}\;{\mathrm{mol}}^{-1}$)	ΔΔ*G*_*elec*_ ($\mathrm{kcal}\;{\mathrm{mol}}^{-1}$)	ΔΔ*G*_*vdW*_ ($\mathrm{kcal}\;{\mathrm{mol}}^{-1}$)
sY1A	4.87 ± 1.16	−0.14 ± 0.61	5.26 ± 1.33
I2A	3.18 ± 0.77	−0.03 ± 0.17	3.18 ± 0.82
sY3A	4.60 ± 1.34	−0.16 ± 0.74	4.88 ± 1.06
T4A	3.78 ± 0.59	3.59 ± 0.58	0.26 ± 0.25
Q5A	−0.70 ± 0.63	−0.02 ± 0.21	−0.83 ± 0.76
sY1Y	1.24 ± 0.27	1.07 ± 0.41	0.35 ± 0.91
sY3Y	1.07 ± 0.74	1.50 ± 1.32	−0.24 ± 0.75

For the isoleucine at position 2nd (PSK^I2^) and threonine at position 4th (PSK^T4^), the alanine-scanning results (see Table 2**)** also indicated reduced binding affinities between mutated PSK peptides and AtPSKR1^LRR^ domain. More specifically, *∆∆G* is calculated to be 3.18 ± 0.77 kcal/mol for PSK^I2A^ mutation that is dominated by the vdW interaction (*∆∆G*_*vdW*_ = 3.18 ± 0.82 kcal/mol) and 3.78 ± 0.59 kcal/mol for PSK^T4A^ one that mainly contributed by electrostatic interaction (*∆∆G*_*elec*_ = 3.59 ± 0.58 kcal/mol). Structural analysis showed that the mutations have negligible effects on the binding interaction networks (Fig. S12), a slight alteration in secondary structures of the antiparallel β-sheet (Fig. S10) and a moderately increased RMSD of AtPSKR1^ID^ (Fig. S11) relative to the WT PSK peptide. Since those two residues PSK^I2^ and PSK^T4^ are nearly fully buried by the AtPSKR1^LRR^ domain (Fig. S8), the decreased residue size (from isoleucine/threonine to alanine) may account for the reduced binding affinities of PSK^I2A^ and PSK^T4A^ mutations. Notably, for the mutation PSK^Q5A^ at position 5th, a slightly enhanced binding affinity (*∆∆G* = −0.70 ± 0.63 kcal/mol) was observed. Moreover, the impact of that mutation on the secondary structure and RMSD of AtPSKR1^ID^ was trivial (Fig. S10 and S11). Therefore, residue PSK^Q5^ can be treated as a good candidate for following design of PSK-like peptides with enhanced binding affinity.

Additionally, we assessed the effect of the sulfate groups of PSK^sY1^ and PSK^sY3^ in directing the binding of PSK peptide. FEP calculations (Table 2) showed that desulfated mutations (PSK^sY1Y^ and PSK^sY3Y^) also result in a weakened binding affinity, with *∆∆G* values of 1.24 ± 0.27 and 1.07 ± 0.74 kcal/mol for PSK^sY1Y^ and PSK^sY3Y^, respectively. Due to the negatively charged nature of sulfate group, the electrostatic interactions (*∆∆G*_*elec*_ = 1.07 ± 0.41 and 1.50 ± 1.32 kcal/mol) were mainly responsible for the reduced binding affinities. As shown in Fig. 3C and F, the desulfated mutations reduced the total number of hydrogen bonds between the PSK peptide and AtPSKR1^LRR^ domain, though some new hydrogen bonds (such as PSK^Y1^ -AtPSKR1^N434^ and PSK^Y3^ - AtPSKR1^N434^) were formed after mutations. Furthermore, unlike the PSK^sY1A^ and PSK^sY3A^ mutations, the secondary structures of AtPSKR1^ID^ in those two desulfated cases (PSK^sY1Y^ and PSK^sY3Y^) are not significantly disrupted.

### 3.4 Rational design of PSK peptide analogs

Searching PSK peptide analogs is crucial for better understanding its signaling role in regulating various cellular processes and for identifying valuable candidates for potential applications in agriculture. Here, encouraged by the above results, we tried to design PSK analogs with enhanced binding affinity through single or double mutations in WT PSK peptide. Due to the critical roles of the two sulfated tyrosines in directing binding, we mainly focused on the other three residues (PSK^I2^, PSK^T4^ and PSK^Q5^). Since residues PSK^I2^ and PSK^T4^ are nearly fully buried by the AtPSKR1^LRR^ domain (Fig. S8), we first checked the hydrophobic variants in those two positions. As shown in Table 3, PSK^I2L^ (ΔΔ*G* = −0.42 ± 0.32 kcal/mol) and PSK^T4I^ (ΔΔ*G* = 0.44 ± 0.84 kcal/mol) mutations had a small influence on the binding affinity, while PSK^I2V^ (ΔΔ*G* = 0.99 ± 0.54 kcal/mol) and PSK^T4V^ (ΔΔ*G* = 1.51 ± 0.69 kcal/mol) moderately weakened the binding affinity, and the phenylalanine mutations at both positions were much unfavorable (PSK^I2F^ with ΔΔ*G* value of 5.75 ± 1.75 kcal/mol and PSK^T4F^ with ΔΔ*G* value of 12.34 ± 1.00 kcal/mol). In addition, the residues with similar physicochemical properties were also explored at site PSK^T4^, and the results were also unfavorable, with ΔΔ*G* values of 2.95 ± 0.77 kcal/mol and 1.87 ± 0.49 kcal/mol for PSK^T4M^ and PSK^T4S^, respectively. Furthermore, a double mutation I2T+T4I *via* swapping the residues of I2 and T4 showed a reduced binding affinity (ΔΔ*G* = 4.23 ± 0.96 kcal/mol). These results declared that residues PSK^I2^ and PSK^T4^ may be already optimal through natural evolution and those two positions are not promising for finding PSK analogs with enhanced binding affinity.

**Table 3:** The binding free energy changes of designed mutations in the PSK peptide calculated by FEP method in PSK-AtPSKR1^LRR^ binding complex.

Mutation	ΔΔ*G* ($\mathrm{kcal}\;{\mathrm{mol}}^{-1}$)	ΔΔ*G*_*elec*_ ($\mathrm{kcal}\;{\mathrm{mol}}^{-1}$)	ΔΔ*G*_*vdW*_ ($\mathrm{kcal}\;{\mathrm{mol}}^{-1}$)
I2L	−0.42 ± 0.32	0.34 ± 0.08	−0.77 ± 0.35
I2V	0.99 ± 0.54	−0.06 ± 0.08	1.05 ± 0.54
I2F	5.75 ± 1.75	0.93 ± 0.32	4.84 ± 1.73
T4M	2.95 ± 0.77	5.34 ± 0.76	−2.27 ± 0.83
T4S	1.87 ± 0.49	0.81 ± 0.62	0.99 ± 0.56
T4V	1.51 ± 0.69	3.04 ± 0.87	−1.71 ± 0.61
T4I	0.44 ± 0.84	0.96 ± 0.47	−0.61 ± 0.80
T4F	12.34 ± 1.00	0.46 ± 0.60	11.86 ± 1.03
Q5L	1.81 ± 0.81	−1.27 ± 0.28	3.33 ± 0.92
Q5V	0.25 ± 0.96	−1.14 ± 0.41	2.26 ± 1.47
Q5Y	0.93 ± 0.87	−0.88 ± 0.49	2.06 ± 1.15
Q5F	2.54 ± 0.79	−0.51 ± 0.79	3.20 ± 1.37
Q5W	1.79 ± 0.66	−0.80 ± 0.72	2.52 ± 0.85
Q5K	**−1.38 ± 0.68**	**−1.03 ± 0.33**	**−0.29 ± 0.79**
I2T+T4I	4.23 ± 0.96	3.67 ± 0.67	0.54 ± 0.68

As aforementioned, PSK^Q5A^ variant could slightly enhance the binding capability (*∆∆G* = −0.70 ± 0.63 kcal/mol), mutations at the PSK^Q5^ site appear to be very attractive. Interestingly, though some mutations (such as PSK^Q5L^, PSK^Q5V^, PSK^Q5F^, *etc.*) were still less favorable, we found that PSK^Q5K^ variant possesses enhanced binding affinity with AtPSKR1^LRR^ domain, with a ΔΔ*G* value of −1.38 ± 0.68 kcal/mol. Free energy decomposition analysis further indicated that PSK^Q5K^ was mostly dominated by the electrostatic interaction. To further validate the enhanced binding ability of PSK^Q5K^ variant, the structural detail after mutation was analyzed in depth and compared to the WT binding complex (Fig. S13). For the PSK^Q5K^ mutation, the hydrogen bonds with AtPSKR1^R310^, AtPSKR1^T335^, and AtPSKR1^N356^ were also kept, and the newly formed electrostatic interactions between positively charged PSK^K5^ residue and the sulfate group of PSK^sY3^ make PSK itself more stable. Then, the stable interatomic distances between PSK^K5^ and PSK^sY3^ in the course of simulations (Fig. S14) prove this point. In short, two designed PSK peptide analogs (PSK^Q5A^and PSK^Q5K^) were predicted to possess enhanced binding affinities with AtPSKR1^LRR^ domain relative to the WT PSK.

### 3.5 Validation of designed PSK analogs by *in vivo* experiments

**Fig. 4:**
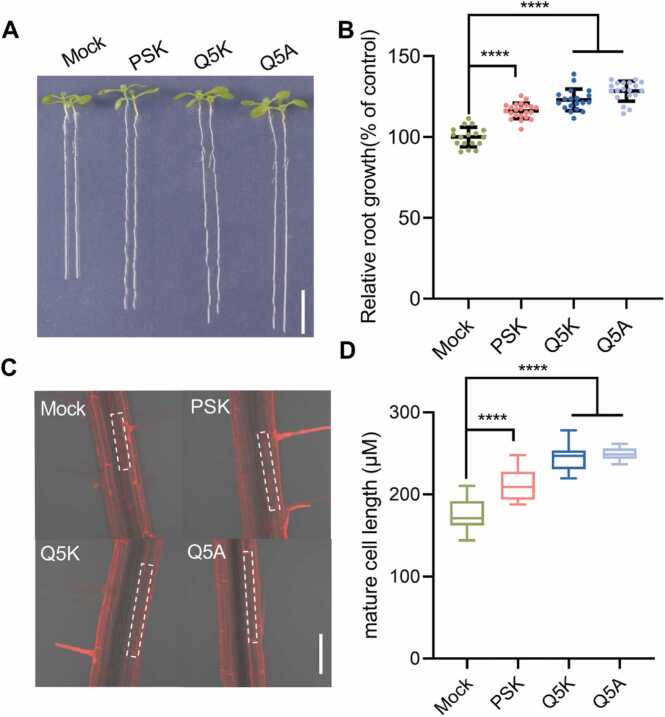
Designed PSK^Q5K^ and PSK^Q5A^ variants can enhance the root growth in *Arabidopsis thaliana*. (A) Root growth of indicated genotypes under control, WT PSK and PSK mutants (0.1 µM) treatment for 8 days (bar = 1 cm). (B) Quantification of relative root growth in (A) (n = 16–20). (C) Confocal images of the seedlings roots in PI staining under control, WT PSK and PSK mutants treatment. Dotted rectangle boxes indicate the outline of mature cell (bar = 100 µm). (D) Measurements of the mature cell length in (C) (n = 15–20). Error bars represent means ± SD. Data were analyzed by ordinary one-way ANOVA (****p < 0.0001). Mock represents untreated control group.

**Fig. 5:**
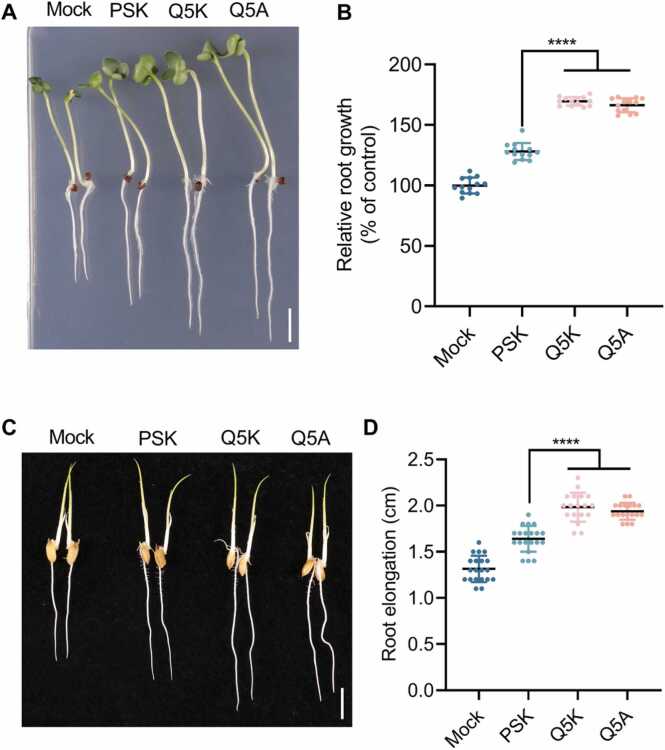
Phenotypic analysis of important crops under PSK and PSK variants treatment. (A) Root growth of Westar under control and PSK (0.2 µM) treatment for 6 days (bar = 1 cm). (B) Quantification of relative root growth in (A) (n = 10). The phenotypes (C) and root elongation (D) of Nipponbare under hydroponic culture in the presence or absence of PSK (0.2 µM) treatment for 24 h (n = 16–20, bar = 1 cm). Error bars represent means ± SD. Data were analyzed by ordinary one-way ANOVA (**** *p* < 0.0001).

The most important biological function of the disulfated pentapeptide PSK is promotion of root growth by enhancing cell expansion [40]. To validate the activities of designed PSK analogs, we conducted experiments *in vivo* to investigate their effects on promoting root growth in *Arabidopsis thaliana*. Two designed PSK analogs were examined, including PSK^Q5A^ and PSK^Q5K^, which were predicted to enhance the binding affinity with AtPSKR1^LRR^ (see Table 2, Table 3). The phenotypical analysis (Fig. 4A-B and Table S3) showed that PSK-mediated root growth promotion was substantially greater in the variant PSK^Q5K^ and PSK^Q5A^ compared to WT PSK peptide treatment. To further investigate this effect, we measured the length of mature *Arabidopsis thaliana* cells of under no treatment, as well as with WT PSK and different PSK variant treatments. (Fig. 4C-D and Table S4). We found that WT PSK treatment significantly increased the mature cell length, with the variants PSK^Q5K^ and PSK^Q5A^ further enhancing this effect. Furthermore, we extended our validation to important crop species by conducting *in vivo* experiments on the monocotyledonous crop *Oryza sativa* and the dicotyledonous crop *Brassica napus* (Fig. 5 and Table S5). Consistent with our findings in *Arabidopsis thaliana*, PSK^Q5K^ and PSK^Q5A^ promoted root growth more effectively than the WT PSK peptide in both crop species. Taken together, these findings strongly demonstrate that Gln-to-Lys and Gln-to-Ala substitutions at the fifth position of PSK detectably increase the root growth promotion, consistent with the FEP calculations.

**Fig. 6:**
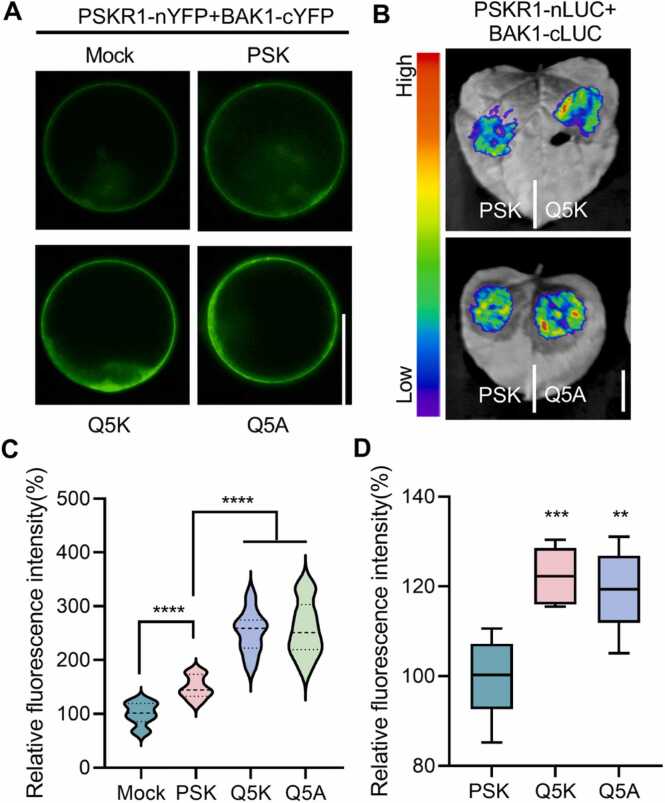
Designed PSK^Q5K^ and PSK^Q5A^ variants can promote the interaction of PSKR1 with BAK1. (A) BiFC mediated detection of interaction between PSKR1 and BAK1 in the *Arabidopsis* mesophyll protoplasts, following control, WT PSK and different PSK variants (2 µM) treatments for 10 min (bar = 20 µm). (B) Split luciferase complementation assay showing the interaction of PSKR1 with BAK1 in the *N. benthamiana* leaves, following WT PSK and different PSK variants (2 µM) treatments for 2 h (bar = 1 cm). (C, D) Relative fluorescence intensity in (A, B) (n = 10), respectively. Error bars represent means ± SD. Data were analyzed by ordinary one-way ANOVA (** *p* < 0.01, *** *p* < 0.001, **** *p* < 0.0001).

Given that the interaction of PSKR1 with SERK1/BAK1 co-receptors is required for PSK signaling [15], we further investigated whether different PSK variants treatments directly affect the interaction between PSKR1 and co-receptor SERK1/BAK1. Bimolecular fluorescence complementation (BiFC) assays (Fig. 6A and C, Table S6) and split-luciferase complementation (LUC) assays (Fig. 6B and D, Table S7) showed that the WT PSK treatment significantly promoted the interaction between PSKR1 and BAK1 compared with the control, and the PSK^Q5K^ and PSK^Q5A^ treatments further accelerated this promotion. These results suggested that PSK^Q5K^ and PSK^Q5A^ peptide analogs can enhance PSKR1-mediated PSK signaling by facilitating the interaction between PSKR1 and co-receptor BAK1, thus promoting root growth.

## 4 Conclusions

Recognition of PSK peptide hormone by PSKR initiates a signaling pathway that regulates various cellular processes in plants, including cell proliferation, differentiation, and growth [13], [14]. In this work, we investigated the structure-property relationship, structural dynamics, and binding property of the PSK peptide with the AtPSKR1^LRR^. Our MD simulations confirmed that the binding affinity and specificity of PSK are contributed by a combination of hydrogen bonds and hydrophobic interactions with the inner side helical structure of AtPSKR1^LRR^ and an anti-β-sheet from the island domain. The FEP results demonstrated that the sulfated tyrosines (PSK^sY1^ and PSK^sY3^) are indispensable for the PSK-AtPSKR1^LRR^ binding complex, while alanine substitution at the PSK^Q5^ site is rather tolerated. Moreover, the PSK peptide analogs were designed to enhance its binding affinity to the AtPSKR1^LRR^ domain by FEP calculations, where the mutants PSK^Q5K^ and PSK^Q5A^ are promising. Our subsequent *in vivo* experiments confirmed that these two designed variants have a stronger promoting effect on stimulating plant root growth than WT PSK. This work reveals novel insights into PSK-AtPSKR1^LRR^ binding mechanism and provides an effective strategy for designing PSK peptide analogs, which has opened up new avenues for the development of peptide-based drugs and biotechnologies that can modulate the signaling pathways involved in plant growth and development. The designed modulators show the potential to improve crop productivity and sustainability, thereby offering a potential basis for alleviating the global food shortage.

## Author statement

The authors declare no competing interest.

## Author contributions

Conception of the study (Ruhong Zhou and Rui Ye). Molecular dynamics (MD) simulations (Rui Ye). Experimental work (Chen Xu). Data processing, analysis and figure preparation (Rui Ye, Chen Xu, Dong Zhang, and Ruhong Zhou). Manuscript writing (Rui Ye, Chen Xu, Dong Zhang, Zhong-Jie Ding, Shao-Jian Zheng, Siewert-Jan Marrink and Ruhong Zhou).

The authors declare no competing interest.

## CRediT authorship contribution statement

**Ruhong Zhou:** Writing – review & editing, Supervision, Resources, Project administration, Funding acquisition, Data curation, Conceptualization. **Dong Zhang:** Writing – review & editing, Supervision, Conceptualization. **Rui Ye:** Writing – original draft, Visualization, Validation, Software, Formal analysis, Data curation, Conceptualization. **Siewert-Jan Marrink:** Writing – review & editing, Supervision. **Shao-Jian Zheng:** Writing – review & editing, Supervision. **Zhong-Jie Ding:** Writing – review & editing, Supervision. **Chen Xu:** Writing – original draft, Visualization, Validation, Investigation, Formal analysis, Data curation.

## Declaration of Competing Interest

The authors declare that they have no known competing financial interests or personal relationships that could have appeared to influence the work reported in this paper.
