# Supplementary material for: Mutagenesis-based optimal design of plant peptide phytosulfokine for enhanced biological activity
Source: Comput Struct Biotechnol J. 2025 Mar 24;27:1296–304. doi: 10.1016/j.csbj.2025.03.029 (PMC11994915; doi:10.1016/j.csbj.2025.03.029)
Supplement: Supplementary file 1 — Supplementary material [file mmc1.docx]

**Supplementary material**

**Mutagenesis-based optimal design of plant peptide phytosulfokine for enhanced biological activity**

Rui Ye^1,3,#^, Chen Xu^1,#^, Zhong-Jie Ding^1^, Shao-Jian Zheng^1^, Siewert-Jan Marrink^3^, Dong Zhang^1,*^, and Ruhong Zhou^1,2,*^

^1^ Institute of Quantitative Biology, School of Physics and College of Life Sciences, Zhejiang University, Hangzhou, Zhejiang 310058, China

^2^ The First Affiliated Hospital, College of Medicine, Zhejiang University, Hangzhou, Zhejiang 310058, China

^3^Groningen Biomolecular Sciences and Biotechnology Institute, University of Groningen, 9747 AG Groningen, The Netherlands

^#^ These authors contributed equally: Rui Ye and Chen Xu

^*^ To whom correspondence should be addressed: [rhzhou@zju.edu.cn](mailto:rhzhou@zju.edu.cn) (R.Z.) and [zhangd_iqb@zju.edu.cn](mailto:zhangd_iqb@zju.edu.cn) (D.Z.)

**
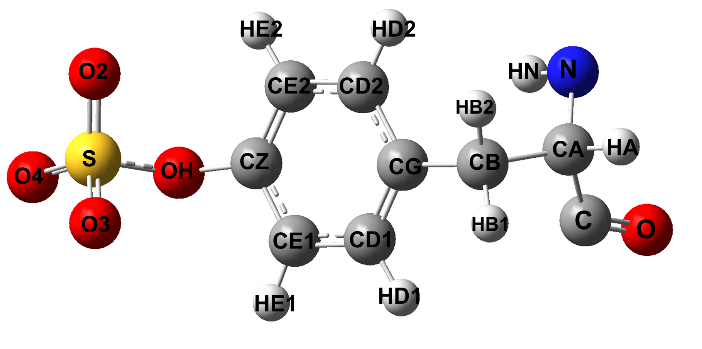
Table S1.** The CGENFF parameters of sY residue.

| Atom name | q (\|e\|) | σ(nm) | $\varepsilon(kJ/mol)$ |
| --- | --- | --- | --- |
| N | -0.47 | 0.32963 | 0.83680 |
| HN | 0.31 | 0.04000 | 0.19246 |
| CA | 0.07 | 0.3563 | 0.13389 |
| HA | 0.09 | 0.23520 | 0.09205 |
| CB | -0.18 | 0.35814 | 0.23430 |
| HB1 | 0.09 | 0.23876 | 0.14226 |
| HB2 | 0.09 | 0.23876 | 0.14226 |
| CG | 0 | 0.35501 | 0.29288 |
| CD1 | -0.135 | 0.35501 | 0.29288 |
| HD1 | 0.115 | 0.24200 | 0.12552 |
| CE1 | -0.192 | 0.35501 | 0.29288 |
| HE1 | 0.115 | 0.24200 | 0.12552 |
| CZ | 0.271 | 0.35501 | 0.29288 |
| OH | -0.431 | 0.31538 | 0.63639 |
| S | 0.929 | 0.37418 | 1.96648 |
| O2 | -0.525 | 0.30291 | 0.50208 |
| O3 | -0.525 | 0.30291 | 0.50208 |
| O4 | -0.525 | 0.30291 | 0.50208 |
| CD2 | -0.135 | 0.35501 | 0.29288 |
| HD2 | 0.115 | 0.24201 | 0.12552 |
| CE2 | -0.192 | 0.35501 | 0.29288 |
| HE2 | 0.115 | 0.24201 | 0.12552 |
| C | 0.51 | 0.35636 | 0.46024 |
| O | -0.51 | 0.30291 | 0.50208 |

**Table S2.** The relative binding free energy changes (ΔΔ*G*) from experiment and simulation (FEP calculations) for five mutations in PSK-DcPSKR^LRR^ binding complex. Mutant residues in the DcPSKR^LRR^ protein are shown in Figure 2B.

| Mutation | Experiment (kcal $\mathbf{mol}^{\mathbf{-1}}$) | Simulation (kcal $\mathbf{mol}^{\mathbf{-1}}$) |
| --- | --- | --- |
| R310A | 1.83 ± 0.11 | 2.57 ± 0.81 |
| T408L | 4.14 ± 0.08 | 4.95 ± 0.88 |
| D455A | 4.33 ± 0.07 | 2.80 ± 0.95 |
| W458A | 2.13 ± 0.10 | 3.13 ± 0.98 |
| F516A | 1.75 ± 0.09 | 0.85 ± 0.55 |

**Table S3.** Raw data of relative root growth in Figure 4A-B.

| Relative root growth (% of control) | | | |
| --- | --- | --- | --- |
| Mock | WT PSK | Q5K | Q5A |
| 104.4365 | 115.2191 | 128.69 | 133.9498 |
| 96.04999 | 115.2775 | 125.5341 | 114.4301 |
| 108.3229 | 113.2028 | 121.0633 | 128.69 |
| 99.9364 | 110.8943 | 111.508 | 118.5795 |
| 91.31616 | 113.4658 | 117.2061 | 124.4237 |
| 108.206 | 120.7711 | 115.5697 | 125.8264 |
| 111.3911 | 118.2289 | 115.5113 | 133.3946 |
| 96.37142 | 119.5438 | 123.9562 | 128.7485 |
| 102.2449 | 120.2159 | 121.2094 | 116.8555 |
| 99.7903 | 114.8392 | 128.6316 | 135.5278 |
| 96.07921 | 104.8163 | 120.8295 | 133.57 |
| 96.13765 | 110.1638 | 126.2062 | 131.6121 |
| 91.75447 | 110.8359 | 119.3685 | 128.9238 |
| 90.84862 | 118.2289 | 118.2289 | 129.5374 |
| 104.1442 | 116.9139 | 123.5471 | 126.2062 |
| 101.4851 | 117.586 | 121.5308 | 131.6121 |
| 101.4851 | 116.1542 | 123.5471 | 133.5407 |
|  | 112.1801 | 135.031 | 135.5278 |
|  | 123.5179 | 138.9174 | 128.3865 |
|  | 118.8425 |  |  |
|  | 125.5049 |  |  |
|  | 119.5146 |  |  |

| Mature cell length (μM) | | | |
| --- | --- | --- | --- |
| Mock | WT PSK | Q5K | Q5A |
| 153.344 | 205.723 | 225.902 | 257.126 |
| 163.671 | 206.435 | 247.248 | 243.622 |
| 144.176 | 198.554 | 230.532 | 245.906 |
| 149.51 | 214.192 | 235.175 | 251.752 |
| 161.859 | 209.371 | 252.349 | 256.77 |
| 173.552 | 211.122 | 254.414 | 256.737 |
| 174.938 | 194.228 | 264.391 | 245.568 |
| 189.714 | 215.311 | 258.79 | 261.93 |
| 177.734 | 225.974 | 252.349 | 236.928 |
| 192.852 | 233.466 | 278.053 | 252.791 |
| 172.486 | 193.695 | 231.207 | 254.181 |
| 205.659 | 236.525 | 253.017 | 245.658 |
| 159.159 | 191.288 | 220.777 | 253.275 |
| 164.142 | 190.979 | 251.011 | 238.719 |
| 210.507 | 204.223 | 236.08 | 244.592 |
| 198.2 | 187.783 | 219.72 | 260.081 |
| 167.497 | 240.022 | 246.615 | 253.854 |
| 164.767 | 248.018 |  | 243.601 |
| 206.953 | 229.917 |  | 237.023 |
| 170.013 | 192.093 |  | 241.441 |
|  | 223.737 |  |  |

**Table S4.** Raw data of mature cell length in Figure 4C-D.

**Table S5.** Raw data of relative root growth in Figure 5.

| Brassica napus relative root growth (% of control) | | | | Oryza sativa root elongation (cm) | | | |
| --- | --- | --- | --- | --- | --- | --- | --- |
| Mock | PSK | Q5K | Q5A | Mock | PSK | Q5K | Q5A |
| 94.35046 | 125.5744 | 167.9981 | 172.7835 | 1.4 | 1.4 | 2.2 | 1.9 |
| 98.76253 | 133.8894 | 165.5885 | 159.1061 | 1.5 | 1.7 | 2.1 | 1.9 |
| 101.2061 | 133.4482 | 173.6999 | 158.597 | 1.4 | 1.7 | 1.9 | 1.8 |
| 89.56506 | 120.4835 | 165.6903 | 161.923 | 1.6 | 1.9 | 2 | 1.9 |
| 105.9576 | 127.4749 | 169.6611 | 157.9182 | 1.3 | 1.4 | 2 | 2 |
| 93.63774 | 129.9185 | 172.7156 | 169.0163 | 1.5 | 1.6 | 1.7 | 2 |
| 111.8969 | 145.6323 | 175.872 | 168.8127 | 1.2 | 1.4 | 2 | 1.8 |
| 107.8242 | 119.1599 | 164.6382 | 172.0029 | 1.1 | 1.8 | 2.1 | 2 |
| 93.19653 | 129.9525 | 172.0029 | 171.9011 | 1.2 | 1.7 | 2.3 | 1.9 |
| 97.64254 | 125.0653 | 171.9011 | 166.0297 | 1.2 | 1.8 | 2 | 2 |
| 98.18557 | 121.3999 | 169.729 | 162.2964 | 1.2 | 1.6 | 2 | 1.9 |
| 101.4437 | 125.9816 | 165.6563 | 169.1181 | 1.3 | 1.6 | 1.7 | 1.9 |
| 106.3309 | 127.3731 | 169.6272 | 173.1569 | 1.1 | 1.8 | 1.9 | 2 |
|  |  |  |  | 1.2 | 1.6 | 1.8 | 2 |
|  |  |  |  | 1.5 | 1.5 | 2.1 | 2.1 |
|  |  |  |  | 1.4 | 1.6 | 1.9 | 2.1 |
|  |  |  |  | 1.4 | 1.6 | 1.9 | 1.9 |
|  |  |  |  | 1.2 | 1.7 | 2.1 | 1.8 |
|  |  |  |  | 1.3 | 1.7 |  |  |
|  |  |  |  | 1.3 | 1.7 |  |  |

| Relative fluorescence intensity (%) | | | |
| --- | --- | --- | --- |
| Mock | WT PSK | Q5K | Q5A |
| 65.41698 | 160.8927 | 227.0058 | 332.3433 |
| 68.8437 | 177.7723 | 319.9773 | 244.897 |
| 91.56309 | 146.8 | 187.2231 | 208.7773 |
| 90.95638 | 130.8402 | 253.9354 | 224.5082 |
| 121.9216 | 141.9652 | 292.0789 | 223.0857 |
| 125.6908 | 177.5865 | 207.6027 | 293.456 |
| 113.8787 | 120.5111 | 230.8208 | 184.9188 |
| 100.2796 | 138.6018 | 266.8022 | 335.6017 |
| 118.5351 |  | 268.7324 | 256.9708 |
| 102.9141 |  | 264.0414 | 277.7993 |

**Table S6.** Raw data of relative fluorescence intensity in Figures 6A and 6C.

**Table S7.** Raw data of relative fluorescence intensity in Figures 6B and 6D.

| Relative fluorescence intensity (%) | | |
| --- | --- | --- |
| WT PSK | Q5K | Q5A |
| 100.2804 | 130.4232 | 105.1202 |
| 85.22968 | 126.6991 | 118.6768 |
| 103.8487 | 116.3949 | 131.0815 |
| 110.6413 | 115.5483 | 122.5789 |
| 100 | 122.2664 | 119.3644 |


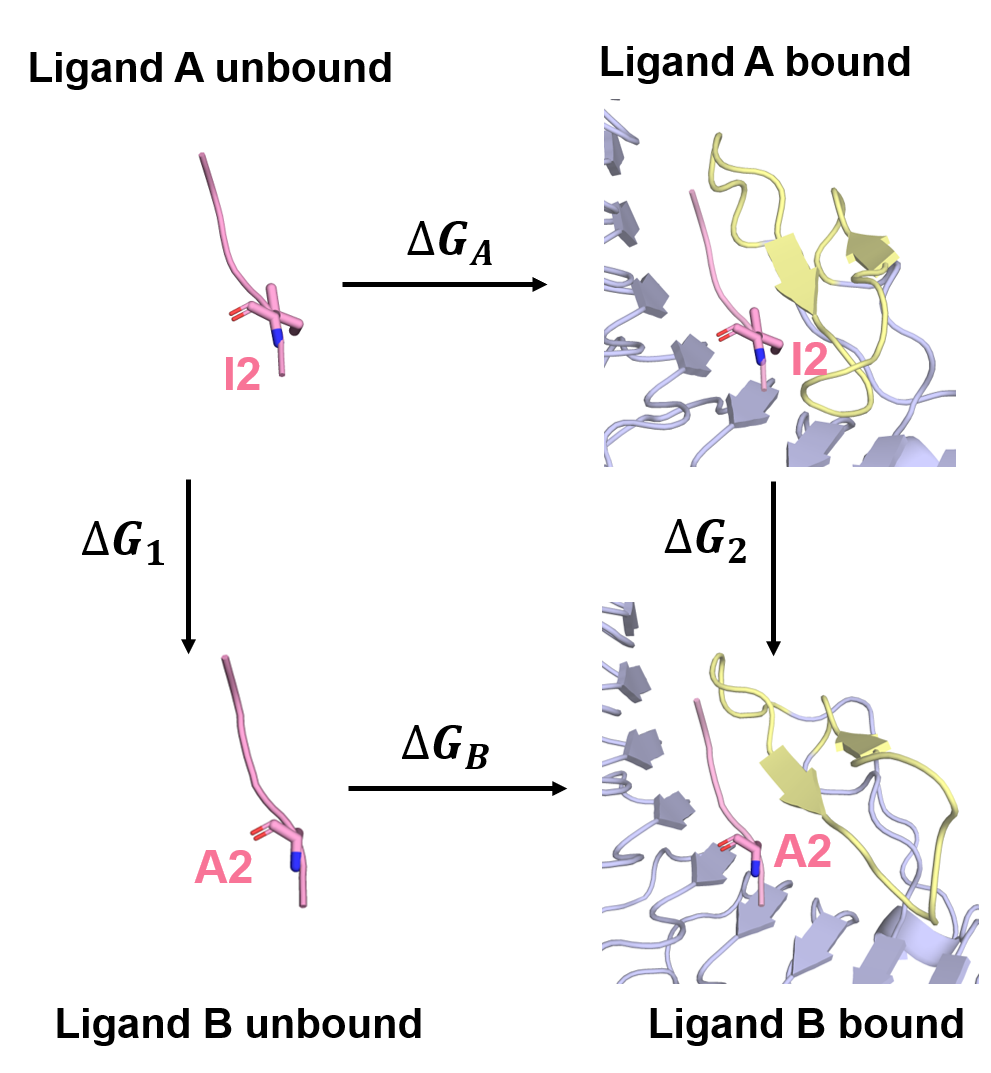


**Figure S1.** Illustration of the thermodynamic cycle for computing the relative binding free energy change ΔΔ*G* of mutating a residue from Ile to Ala (PSK^I2A^). The definitions of each ΔΔ*G* component are listed in the main text.


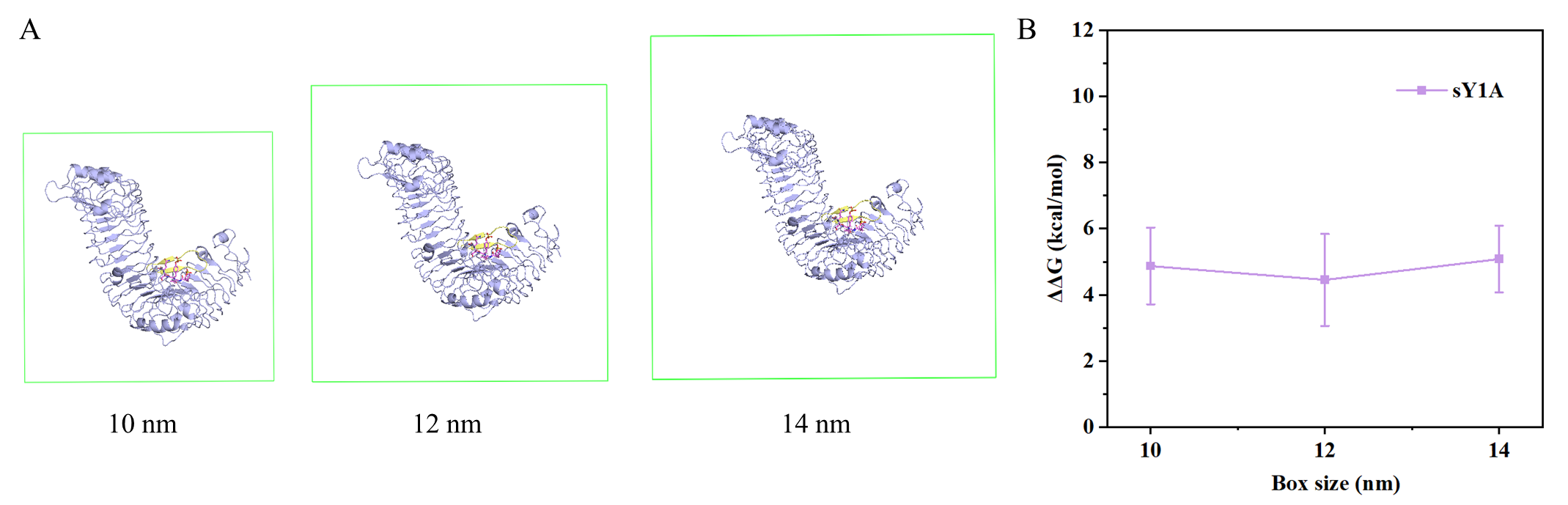


**Figure S2.** (A) Three different simulation boxes in which the PSK-AtPSKR^LRR^ bound complex was solvated by cubic water boxes of different lengths. (B) The calculated relative binding free energy changes for the sY1A mutation in different simulation boxes.


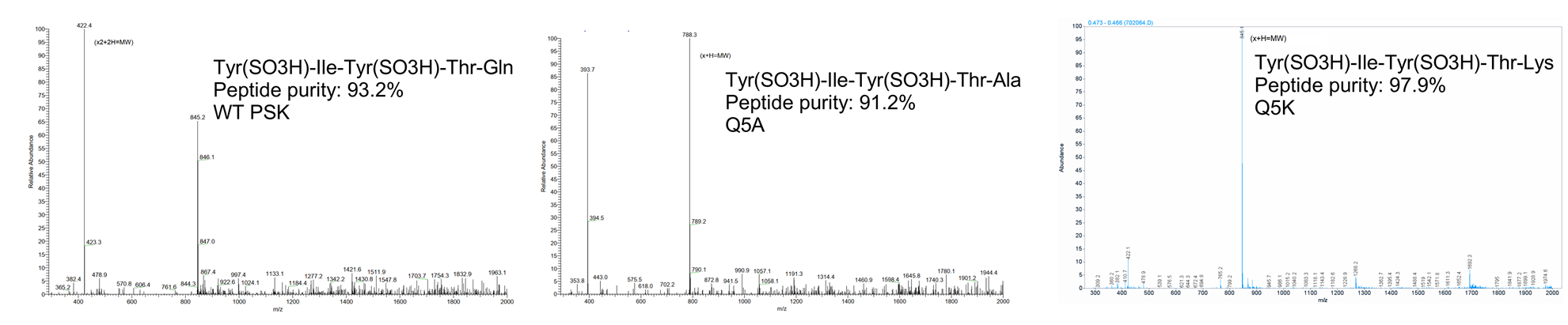


**Figure S3.** MS spectrogram of the peptides.


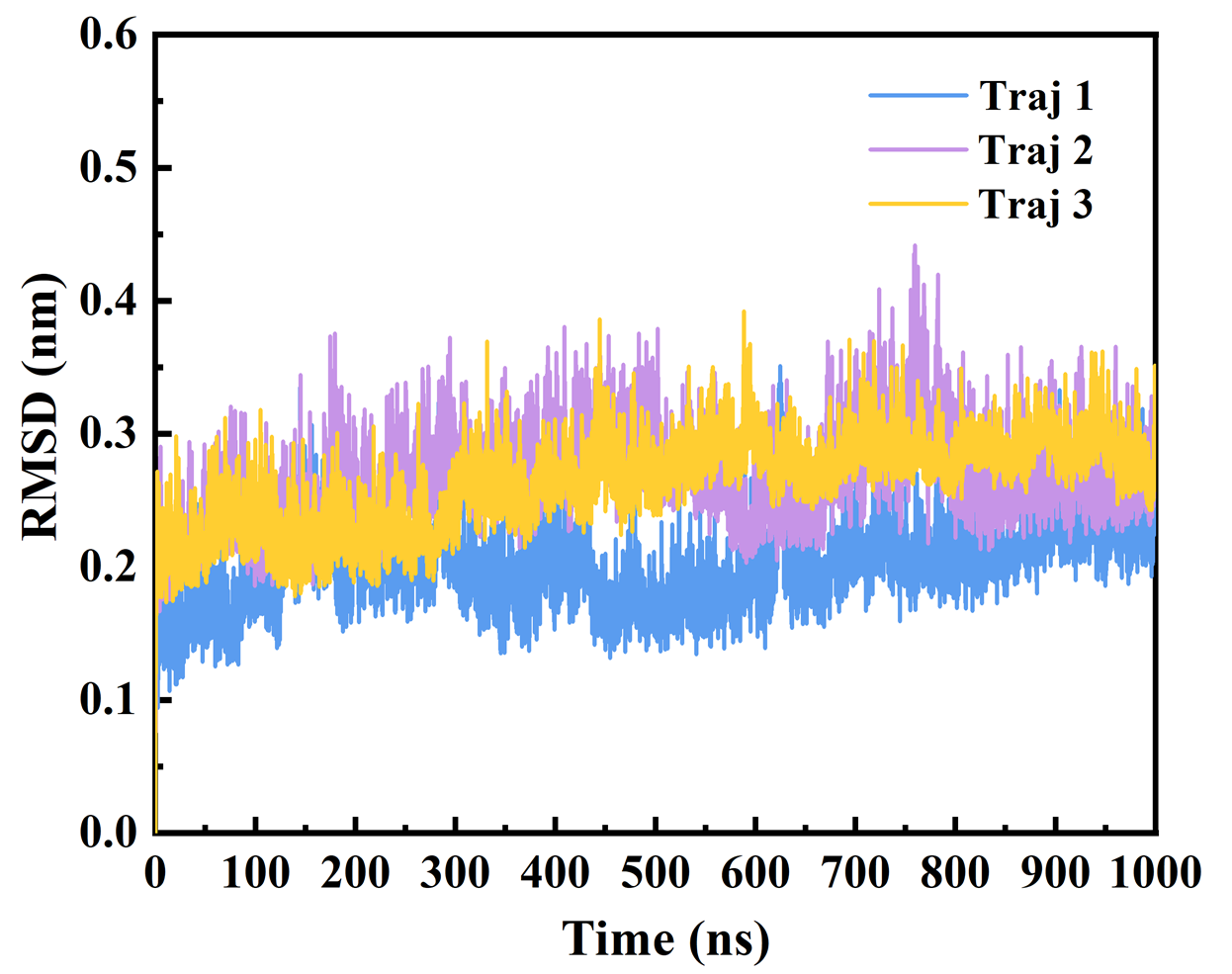


**Figure S4.** Heavy-atom RMSD as functions of simulation time for constructed binding model of PSK and AtPSKR1^LRR^.


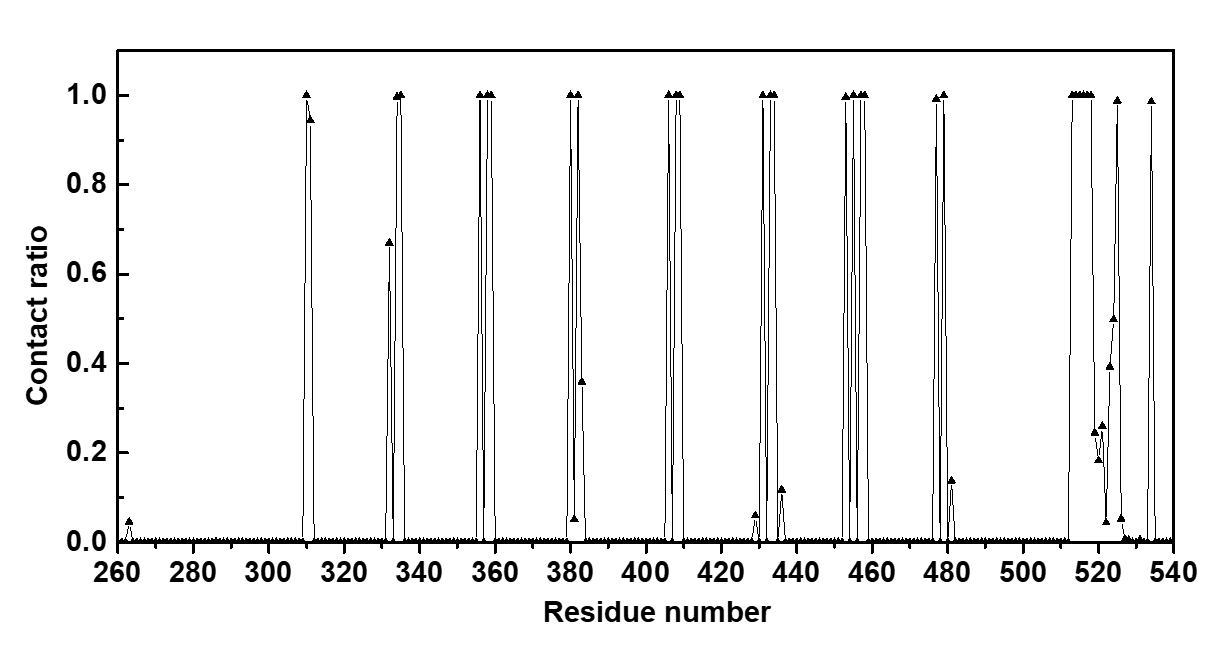


**Figure S5.** The contact ratio of residues of PSK peptide with AtPSKR1^LRR^.

**
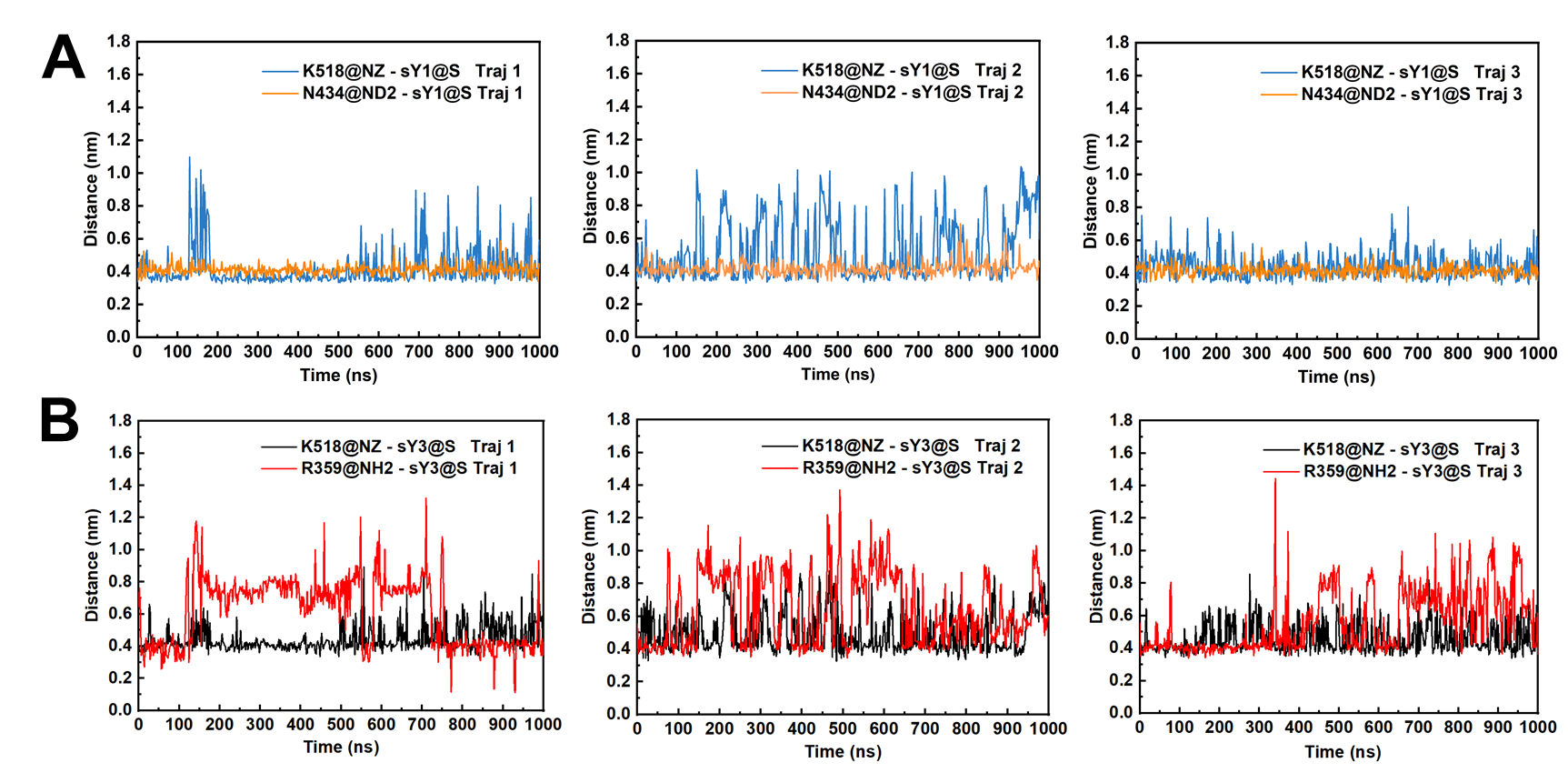
**

**Figure S6**. (A) The distance between atoms on the N-terminal sulphated tyrosine (sY1) and AtPSKR1^LRR^ (K518, N434) during the simulation time. (B) Distances between atoms on the C-terminal sulphated tyrosine (sY3) and AtPSKR1^LRR^ (K518, R359) during the simulation time.


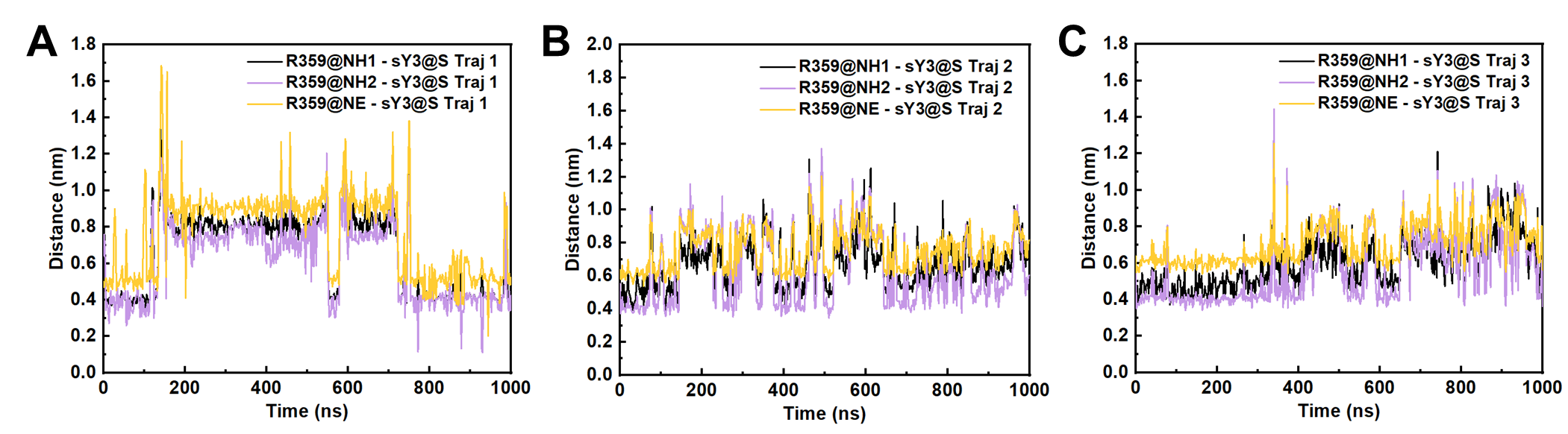


**Figure S7.** Distances between the atom sY3@S and the three arginine atoms of R359 (NH1, NH2 and NE) as functions of simulation time in different MD trajectories.


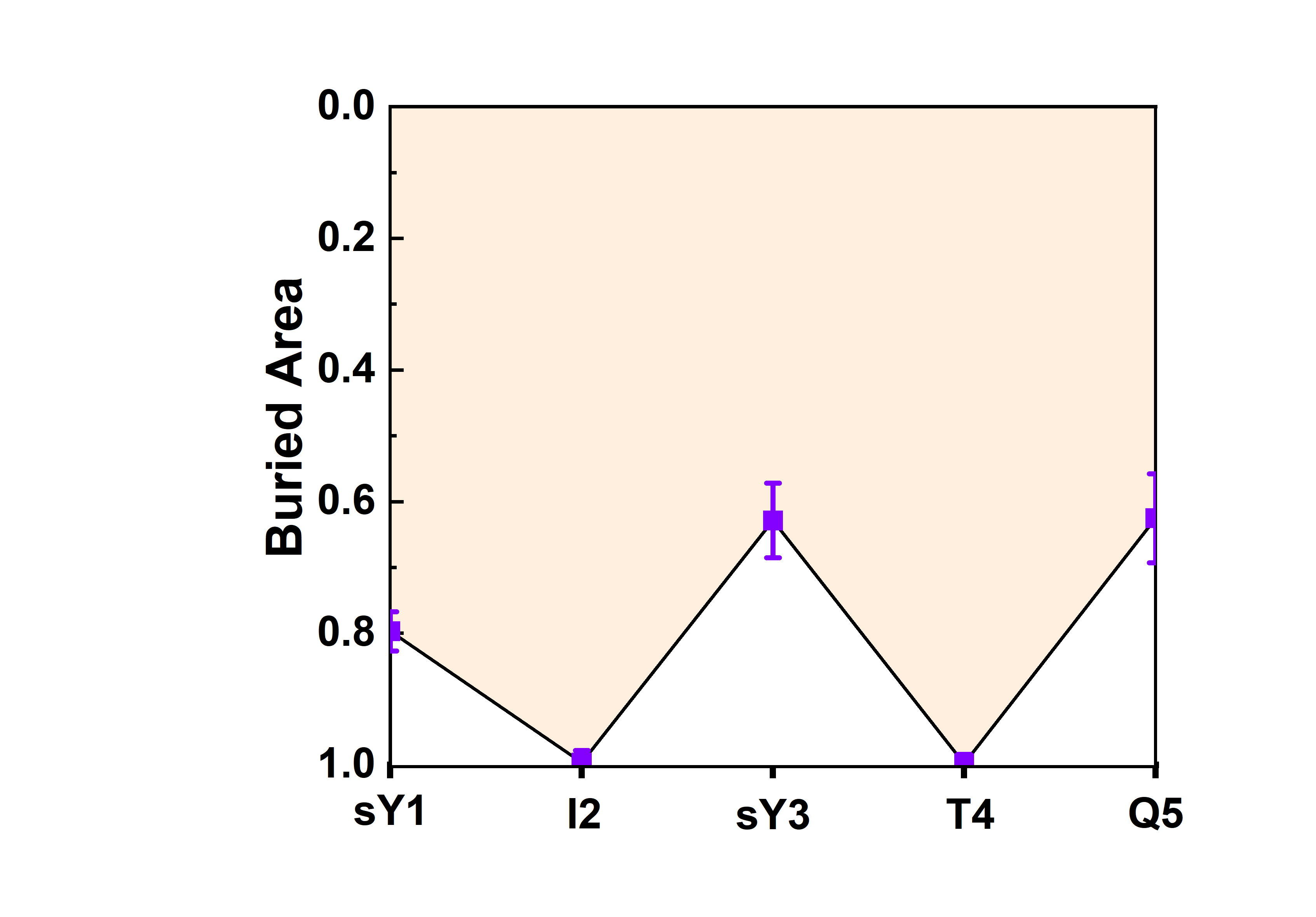


**Figure S8.** Average buried surface area of the PSK peptide in the PSK-AtPSKR1^LRR^ complex extracted from the last 100 ns of three independent MD simulations.


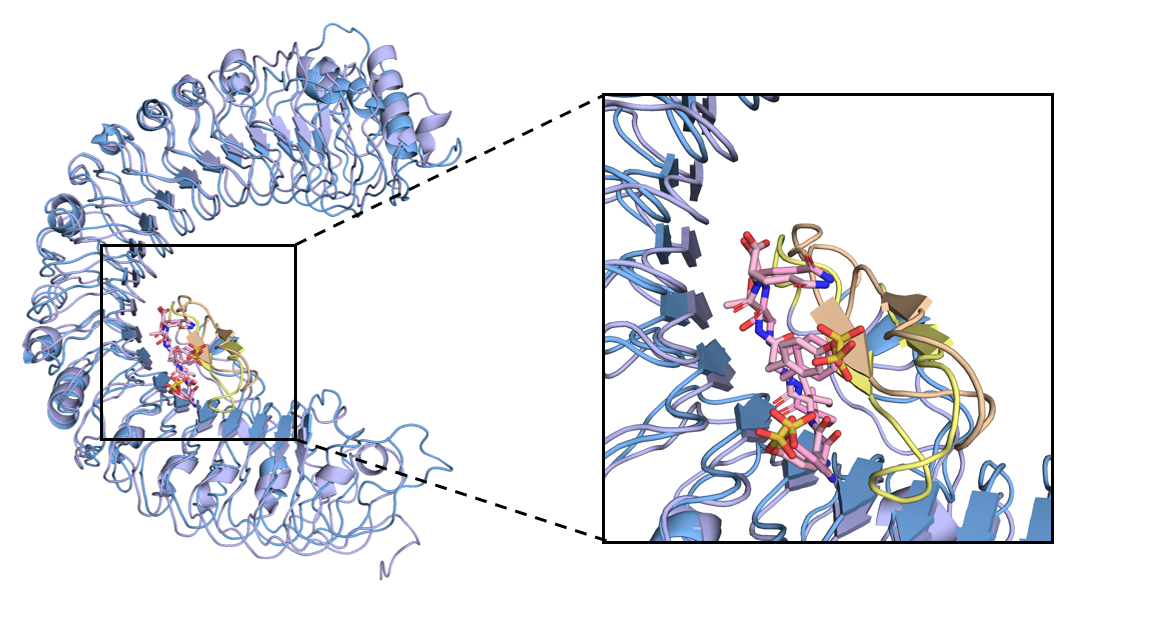


**Figure S9**. Structure comparison of the PSK-DcPSKR^LRR^ complex (pink for PSK and sky-blue for DcPSKR^LRR^) and PSK-AtPSKR1^LRR^ complex (pink for PSK and purple for AtPSKR1^LRR^).


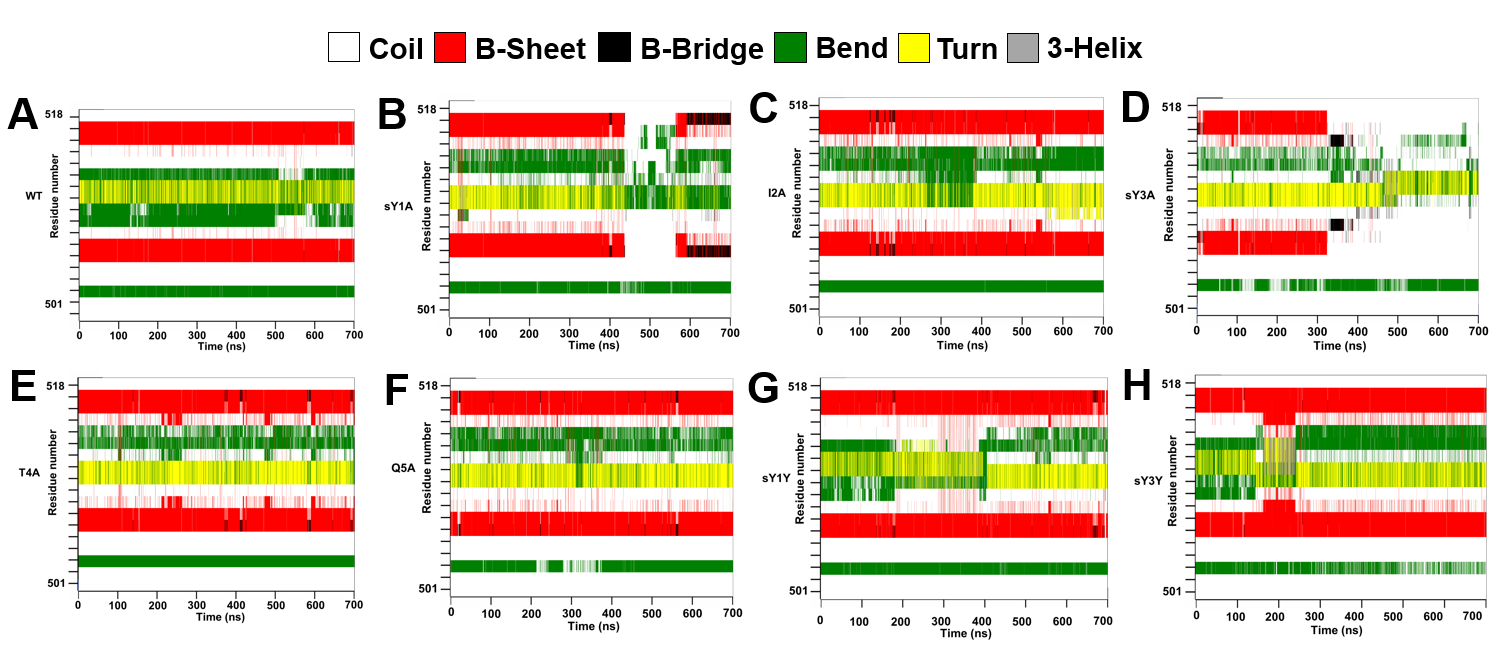


**Figure S10.** The secondary structure of the island domain of AtPSKR1^LRR^ (AtPSKR^ID^) changes over total simulation time in representative trajectories for (A) WT PSK peptide, (B) mutant PSK^sY1A^, (C) mutant PSK^I2A^, (D) mutant PSK^sY3A^, (E) mutant PSK^T4A^, (F) mutant PSK^Q5A^, (G) mutant PSK^sY1Y^, and (H) mutant PSK^sY3Y^.


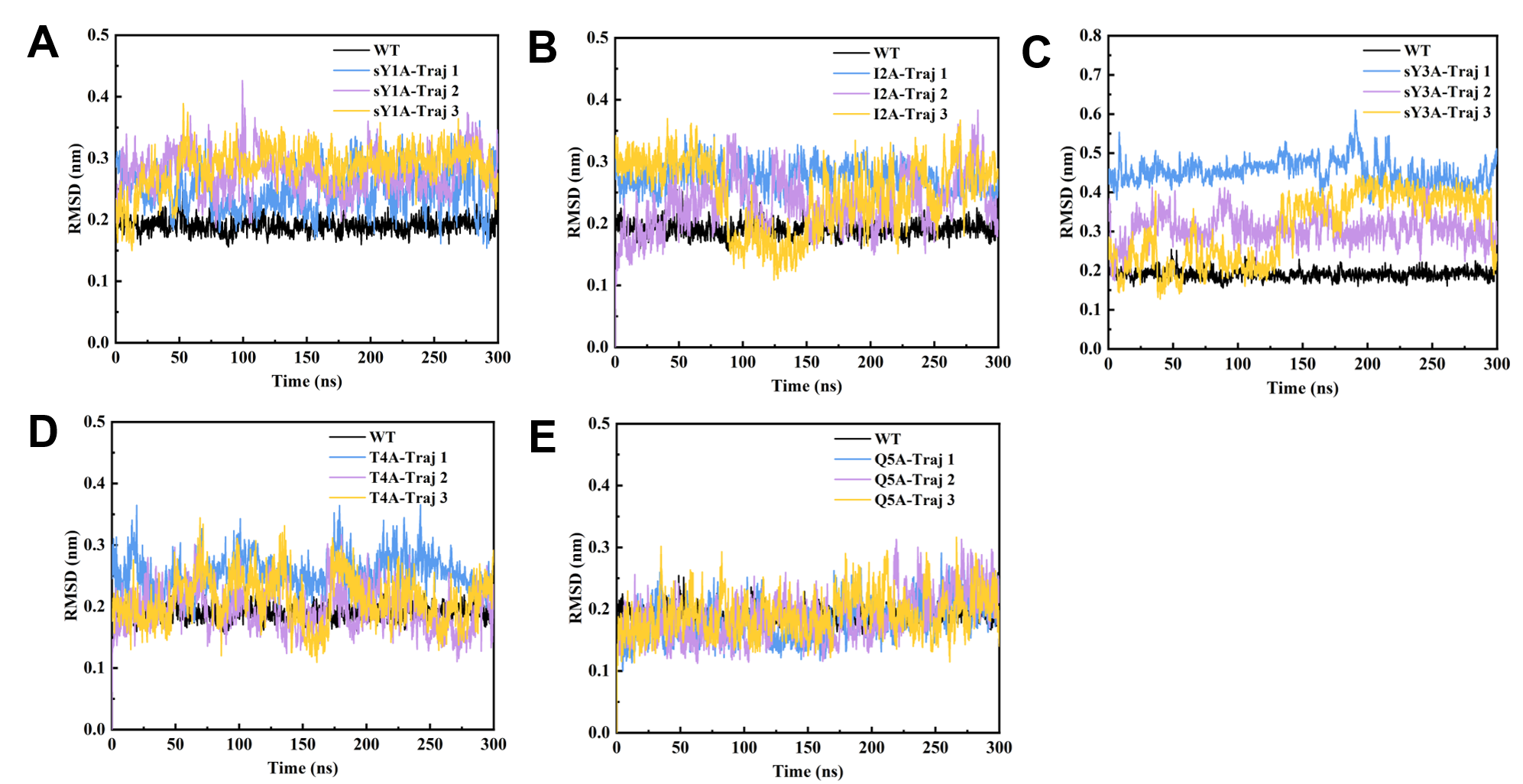


**Figure S11.** Comparison of the RMSDs of the heavy-atom of AtPSK1^ID^ for WT PSK peptide and its mutations: (A) PSK^sY1A^, (B) PSK^I2A^, (C) PSK^sY3A^, (D) PSK^T4A^, and (E) PSK^Q5A^.


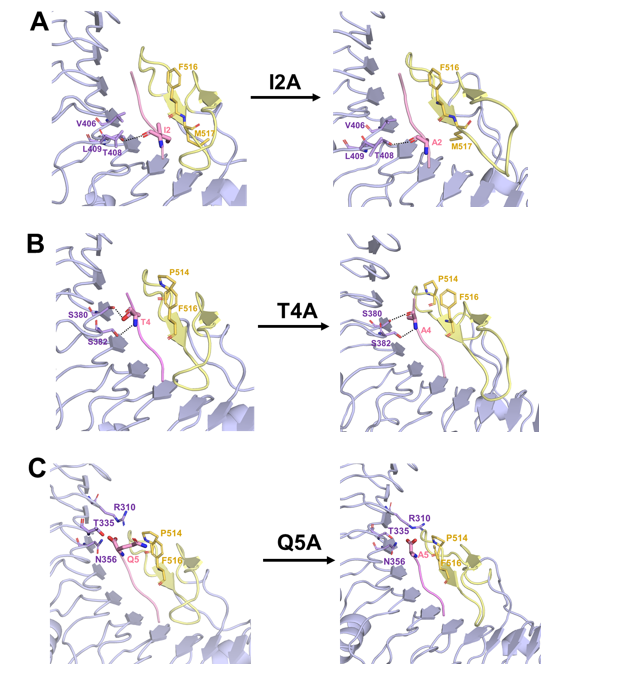


**Figure S12.** Structural comparison of AtPSKR1^LRR^ bound to WT PSK peptide (left) and mutated PSK peptide’s (right) at the end of MD simulations: (A) for PSK^I2A^ mutation; (B) for PSK^T4A^; (C) for PSK^Q5A^.


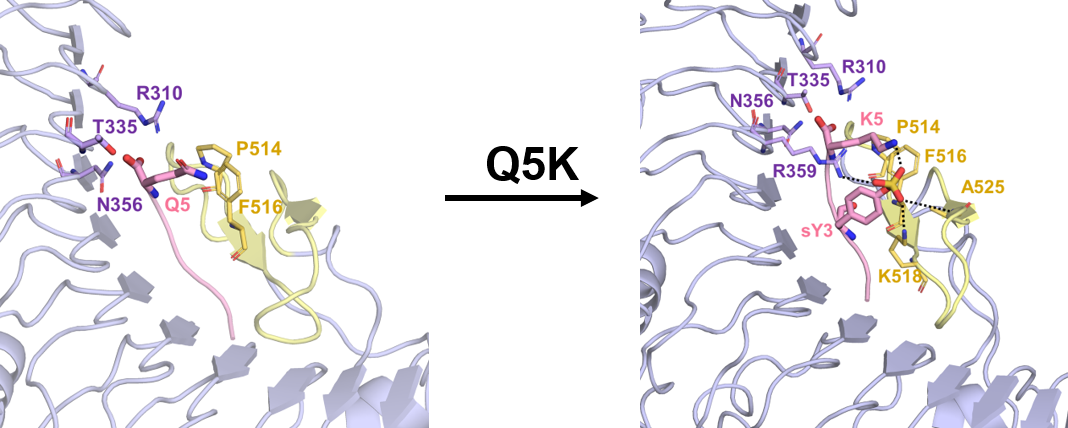


**Figure S13.** Structural comparison of AtPSKR1^LRR^ bound to WT PSK peptide (left) and mutated PSK^Q5K^ peptide (right) at the end of MD simulations.


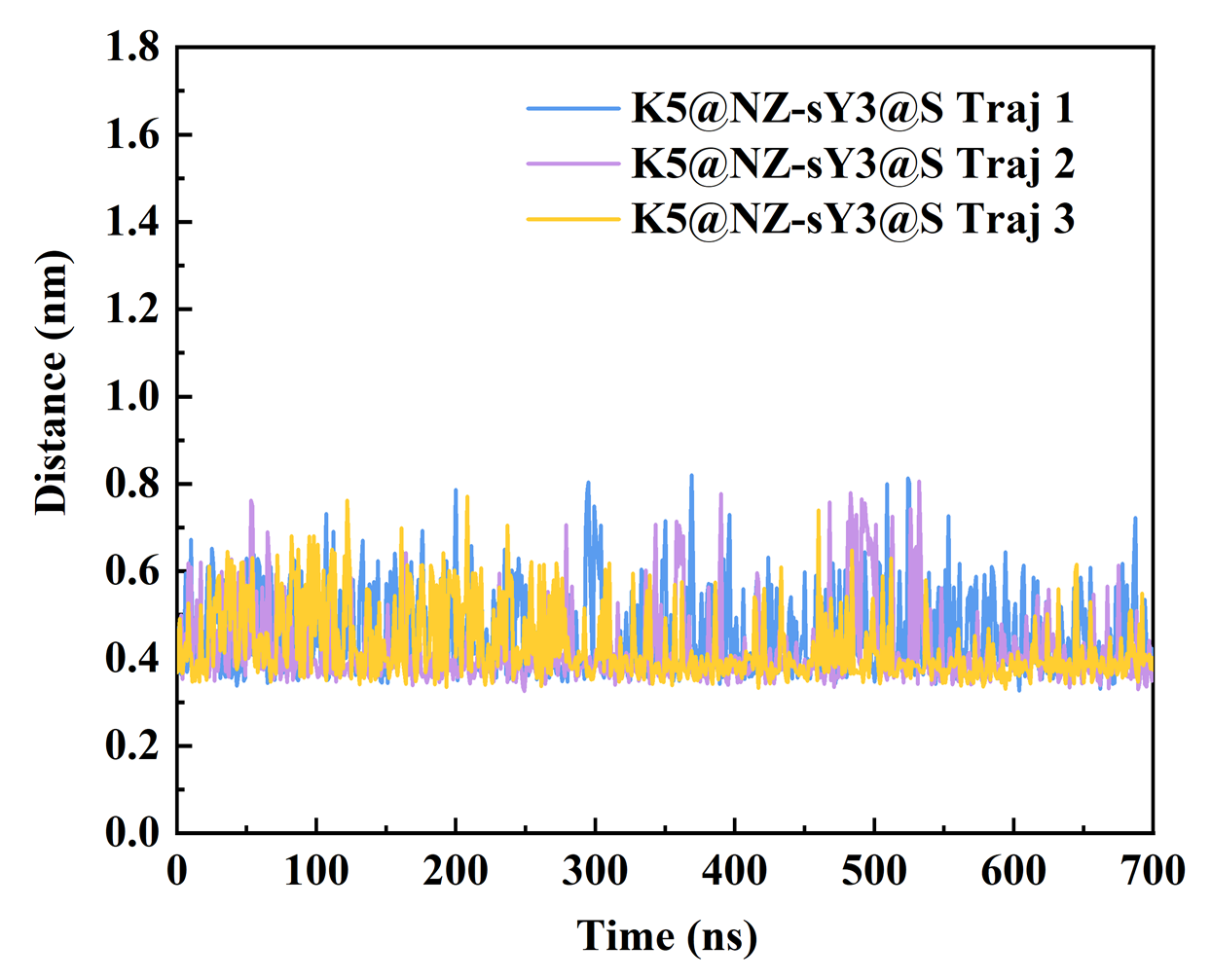


**Figure S14.** Distances between atoms of PSK^K5^ with PSK^sY3^ during the MD simulations.
